# Engineering the Optical Emission and Robustness of Metal‐Halide Layered Perovskites through Ligand Accommodation

**DOI:** 10.1002/adma.202008004

**Published:** 2021-03-01

**Authors:** Balaji Dhanabalan, Giulia Biffi, Anna Moliterni, Vincent Olieric, Cinzia Giannini, Gabriele Saleh, Louis Ponet, Mirko Prato, Muhammad Imran, Liberato Manna, Roman Krahne, Sergey Artyukhin, Milena P. Arciniegas

**Affiliations:** ^1^ Istituto Italiano di Tecnologia Via Morego 30 Genova 16163 Italy; ^2^ Dipartimento di Chimica e Chimica Industriale Università degli Studi di Genova Via Dodecaneso, 31 Genova 16146 Italy; ^3^ Istituto di Cristallografia CNR Via Amendola, 122/O Bari 70126 Italy; ^4^ Paul Scherrer Institute Forschungsstrasse 111 Villigen PSI 5232 Switzerland

**Keywords:** 2D layered perovskites, optical efficiency, organic cations, photoluminescence, white emission

## Abstract

The unique combination of organic and inorganic layers in 2D layered perovskites offers promise for the design of a variety of materials for mechatronics, flexoelectrics, energy conversion, and lighting. However, the potential tailoring of their properties through the organic building blocks is not yet well understood. Here, different classes of organoammonium molecules are exploited to engineer the optical emission and robustness of a new set of Ruddlesden–Popper metal‐halide layered perovskites. It is shown that the type of molecule regulates the number of hydrogen bonds that it forms with the edge‐sharing [PbBr_6_]^4‐^ octahedra layers, leading to strong differences in the material emission and tunability of the color coordinates, from deep‐blue to pure‐white. Also, the emission intensity strongly depends on the length of the molecules, thereby providing an additional parameter to optimize their emission efficiency. The combined experimental and computational study provides a detailed understanding of the impact of lattice distortions, compositional defects, and the anisotropic crystal structure on the emission of such layered materials. It is foreseen that this rational design can be extended to other types of organic linkers, providing a yet unexplored path to tailor the optical and mechanical properties of these materials and to unlock new functionalities.

## Graphical abstract

The organic component in 2D layered perovskites can grant access to many possibilities for material design, in view of the rich variety. This work shows how the choice of the structure and chain length of organic cations can effectively guide the precise design of the emission color and efficiency of such versatile nanomaterials.

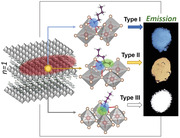

## 1 Introduction

Two‐dimensional (2D) layered perovskites are an attractive platform to design optoelectronic materials.^[^ ^1^, ^2^ ^]^ In particular, the structures made of intercalated organic layers interacting through van der Waals forces and inorganic slabs in the Ruddlesden–Popper layered perovskites (RPLP) offer exciting opportunities for structural engineering by the diverse choices of chemical composition,^[^ ^3^ ^]^ and thus, interlayer spacing, and lattice distortions. Ultimately, a controlled variation of these parameters could effectively guide the tailoring of the electronic band structure of RPLP semiconductors. In this context, metal‐halide RPLP are of particular interest, as they manifest unique properties, such as quantum and dielectric confinement,^[^ ^4^ ^]^ narrow‐ and broadband emission,^[^ ^5^, ^6^ ^]^ fast radiative recombination,^[^ ^7^ ^]^ long carrier diffusion lengths,^[^ ^8^ ^]^ and large exciton binding energies.^[^ ^9^ ^]^ These properties come along with low‐cost processing and simplified device architectures. Besides, the presence of the organic layers provides structural stability and protection from moisture, making them more robust against environmental conditions, which is critical for device performance and lifetime.^[^ ^10^ ^]^

Due to the stacking configuration of RPLP—with relatively strong organic‐inorganic interconnectivity—the type of organoammonium molecule plays a fundamental role in their photophysics.^[^ ^11^ ^]^ For example, by introducing phenethylammonium in the Pb–Br system, the resulting RPLP can show four times higher emission efficiency in the deep blue region compared to butylammonium, a behavior that is related to the high lattice rigidity provided by Pb–Br–π stacking and reduced electron–phonon interaction in the phenethylammonium‐based structures.^[^ ^12^, ^13^ ^]^ Moreover, using longer organic molecules such as decylammonium and dodecylammonium increases the bandgap of the RPLP with respect to materials with shorter molecules in the same family.^[^ ^14^ ^]^ Interestingly, the organoammonium layers can further deform the inorganic slabs and generate both intra‐ and inter‐octahedral distortions that, together with strong exciton–lattice coupling, enable broadband and white emission at room temperature from RPLP.^[^ ^15^, ^16^, ^17^ ^]^ These properties have been recently observed from optically emissive 1‐ and 0D hybrid organic–inorganic metal halides^[^ ^5^, ^18^ ^]^ and all‐inorganic perovskites.^[^ ^19^, ^20^ ^]^ As sources of white light, RPLP display tunable emission and high color rendering index (CRI).^[^ ^5^, ^21^ ^]^ Recent studies report more examples of bluish‐white and bright white emitting RPLP, which are mostly investigated through halide ion modifications (i.e., Cl^−^, Br^−^, and I^−^)^[^ ^22^, ^23^ ^]^ and by using diverse organic molecules.^[^ ^17^, ^24^, ^25^, ^26^, ^27^ ^]^ In general, RPLP have a (100)‐oriented structure that typically exhibits a narrow emission in the deep blue region, with few cases showing white emission; or they can have a (110) crystal orientation (so called corrugated structures), which display cold, warm, or pure white CRI.^[^ ^2^, ^6^, ^17^, ^27^, ^28^ ^]^ Current challenges include control over layer thicknesses, thermal stability, reduced lattice defects and self‐quenching, to improve their so far limited quantum efficiency (≈9%^[^ ^23^ ^]^ from white‐light‐emitting bulk single crystals, which has been increased up to 12%^[^ ^29^ ^]^ from microplates of less than 2 µm lateral size obtained through a slow crystallization in toluene). In view of the rich variety of organic molecules that is available, the material design possibilities toward optoelectronic technologies have only been explored to a very small extent. Therefore, there is an urgent need of rational design criteria enabling precise color tunability in RPLP and, at the same time, satisfying specific requirements (e.g., chromaticity coordinates, color temperature) for diverse applications, which demand control over RPLP architecture while preserving easy processability.

**Figure 1:**
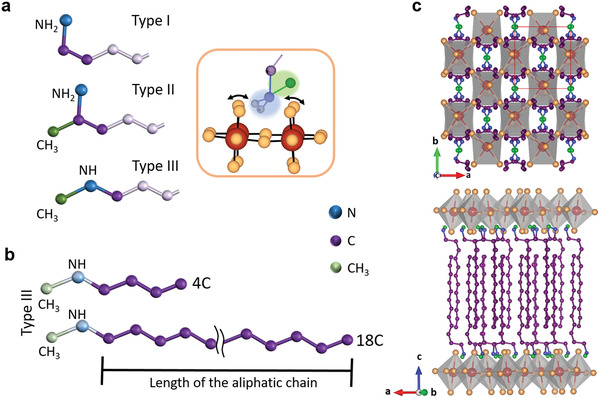
Structure and bonding of different organoamines for RPLPs. a) Representation of the amines used in the synthesis for RPLP structures selected based on their amine bonding group (in blue). Type I, an N—H_2_ terminal group; type II, an N—H_2_ terminal group with an additional methyl group (in green) linked to the aliphatic chain; and type III, an N—H terminal group linking a methyl group and an aliphatic chain (in purple). The aliphatic chains are shown with only some segments in light purple for clarity. b) Type III amines with aliphatic chains of different length (in purple), from 4 to 18 C atoms. In (a) and (b), the sections of interest are displayed in bright colors. c) Representative RPLP structure of the platelets (type III, 12 C) derived from synchrotron single crystal diffraction data collected at 100 K. It shows interdigitated pairs of organoammonium cations packed between two inorganic [PbBr_4_]^2−^ layers. The sites for accommodating the methyl and amino group lie between four Pb–Br octahedra (anchor site) and is highlighted in the orange‐framed inset in (a). Hydrogen atoms are omitted for clarity in (a–c), and only displayed for those on the nitrogen head in the inset of (a).

In this work, we comprehensively investigate RPLP made from organoammonium molecules of different types and elucidate the key role of hydrogen bonding on the lattice dynamics and the light emission of structures with a general formula (Rm)_2_A_*n*−1_Pb_*n*_Br_3*n*+1_ where Rm^+^ is a large organic cation, A is a smaller cation (e.g., Cs^+^, MA^+^ (methylammonium), or FA^+^ (formamidinium)), Br is the selected halogen, and *n* represents the number of inorganic layers separated by the Rm cations. In our case, all the structures are made of single Pb–Br layers (*n* = 1), thus without the presence of small *A* cations. We introduce a new set of pure white emitting RPLP platelets with thicknesses in the range from tens to hundreds of nanometers and micrometer‐sized lateral dimensions. We approached their design in a broad manner through two molecular descriptors: the local structure of the binding group, and the overall length of the molecule. For the local structure of the binding group, we selected three options: i) a primary ammonium group, carrying a linear alkyl chain (**Figure** **1**a, top), named as type I; ii) a primary ammonium group carrying a branched alkyl chain, with a methyl group bound to the carbon α (i.e., directly bound) to the ammonium group (Figure 1a, middle), named as type II; iii) a secondary ammonium group, carrying a methyl group and a linear alkyl chain (Figure 1a, bottom), named as type III. The local structure of the binding group affects the orientational freedom of the molecules at the anchoring site, and the molecule length determines the robustness of the whole structure. We observe narrow deep‐blue emission when using type I molecules. On the other hand, the presence of a methyl group on the carbon α to the ammonium group (type II) largely suppresses the blue emission: in this case the crystals display a broad photoluminescence (PL) that is centered at 590 nm with ≈200 nm full‐width at half‐maximum (FWHM). For type III molecules, the presence of the methyl group attached to the ammonium group affects the hydrogen bonds that can be formed by the organoammonium with the Pb–Br layer and the orientation of the organic cation, leading to substantially different emission spectra from the resulting RPLP. In this case, the emission is characterized by a narrow blue peak and by a broadband emission (throughout the visible) with an amplitude similar to that of the narrow emission peak. In this case the broadband emission is centered at 525 nm. The various structures of the organoammonium cations therefore enable tuning of the color coordinates, from cold to warm to pure white light with CRI above 86. For the type III structures, we also investigated the effect of the long aliphatic chain length on the platelet formation, robustness, and emission properties. The aliphatic chain length has no significant impact on the spectral profile of the PL, but it strongly influences the emission intensity and quantum efficiency, which points to a correlation of the rigidity of the molecular linking in the organic layer to the ability to passivate the surfaces of the inorganic layers. Our findings provide a set of guidelines for the design of RPLP with specific optoelectronic properties for the yet emerging field of solid‐state lighting from single‐layer materials, which require atomic‐level understanding. Future work will extend these structural criteria to other organic molecules used as building blocks to add an extra functionality (i.e., conductive, foldable, dual emission) to the RPLP and to target switchable 2D structures.

## 2 Results and Discussion

### 2.1 Effect of the Organoammonium Bonding Group at the Anchor Site

The use of relatively simple organic molecules and ecofriendly solvents in synthetic protocols performed at room temperature provides a straightforward route to produce RPLP at low cost and low environmental impact. Here we carefully selected three types of organoamines with different bonding group and fabricated RPLP structures with single Pb–Br octahedra layers (*n*  =  1) by following our previously reported protocol^[^ ^30^ ^]^ with slight modifications (see Experimental Section for details). Note that we did not use MA, Cs, or FA precursors or any other source of cations^[^ ^31^, ^32^ ^]^ in the synthesis to avoid the formation of structures with a larger number of adjacent octahedra layers. Figure 1a displays the three types of molecules and Table S1, Supporting Information, summarizes the different amines explored in this work. We refer as type I to those molecules with an N—H_2_ head group—linear primary amines; type II to those with a methyl ramification on the terminal C at the N—H_2_ head group; and type III to those with an N—H head group with a methyl group and the aliphatic chain as substituent groups. We found that for the type III amines a minimum of ten carbons in the aliphatic chain is needed to form RPLP platelets. To decouple the effect of the bonding group from effects of different chain length, we focus first on molecules with ten carbon atoms in their longest alkyl chain (Table S1, Supporting Information): namely, 1‐undecylamine, UDA (type I), 1‐methyldecylamine, 1‐MDA (type II), and *N*‐methyldecylamine, *N*‐MDA (type III). The choice of using UDA in our analysis instead of decylamine, which shows similar characteristics (Figure S1, Supporting Information), was based on its equal number of total carbon atoms as compared to the selected type II and type III amines. The surface analysis performed on the three selected samples via X‐ray photoelectron spectroscopy (XPS) shows that they are characterized by similar spectra (Figure S2, Supporting Information). In all the cases, the N 1s spectrum can be described by a single component centered at 401.7–401.8 eV, which is consistent with the presence of protonated ammonium species (either primary or secondary), and the recorded signals for Pb and Br are at positions typical for Pb(II) and bromides, respectively.^[^ ^33^ ^]^ These positions are also close to those reported from MAPbBr_3_ crystals,^[^ ^34^ ^]^ indicating a similar chemical environment and thus supporting the formation of a perovskite‐like inorganic layer. We expect that the presence of an additional group, which needs to be accommodated between corner‐sharing octahedra, will lead to strong distortions of the Pb–Br octahedra layer, as illustrated in the orange‐framed panel in Figure 1a. Note that most of the RPLP reported so far come from type I organoamines. In some cases, an aromatic ring is attached to a short alkyl chain, which leads to slight changes in color coordinates and distance between Pb–Br slabs (*d*‐spacing).^[^ ^12^, ^30^, ^35^ ^]^ Type III organoamines have been rarely studied in 2D layered perovskites,^[^ ^36^ ^]^ however, they have been used to produce efficient and thermally stable solar cells when combined with 3D perovskites^[^ ^37^ ^]^ and to fabricate bright light‐emitting nanocubes.^[^ ^38^ ^]^ The next question we formulate for this type of molecules is the role of the aliphatic chain length (see Figure 1b) concerning the robustness and light emission of the resulting RPLP. A representative architecture of the RPLP derived from single crystal X‐ray diffraction is displayed in Figure 1c, with the alternated organic‐inorganic layers (tilted c‐view on the bottom panel) and the organoammonium cations filling the void between four Pb–Br octahedra (top panel). The details on the atomic coordination are reported in Tables S3,S4, Supporting Information. To facilitate reading, we label our samples in the following with the bonding group type and number of carbons in their aliphatic chain; their full names can be found in Table S1, Supporting Information.

**Figure 2:**
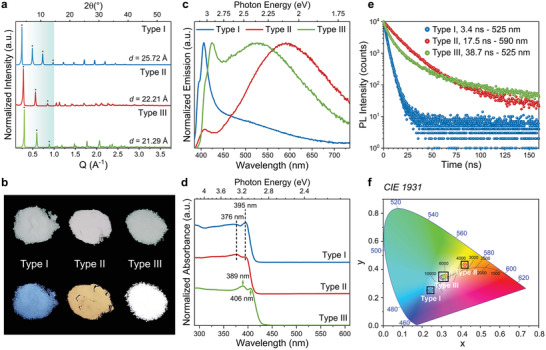
Role of different types of organic cations on the structural and optical properties of RPLPs. a) X‐ray diffraction patterns collected from the type I (UDA)_2_PbBr_4_, type II (1‐MDA)_2_PbBr_4_, and type III (N‐MDA)_2_PbBr_4_, platelets made of single inorganic layers. All the resulting structures show periodic diffraction peaks with reflections associated to the (0 0 2*l*) diffraction orders (noted by the asterisks). The resulting *d*‐spacing values are reported. b) Deposits of the studied platelets seen under day (top) and ultraviolet light (bottom). c,d) Emission and absorption spectra collected from the RPLP crystals. e) PL decay curves acquired at the maxima of broadband emission of the samples, as indicated in the legend. The calculated τ_avg_ values for each sample are reported in the legend along with the type of amine used in the synthesis. f) CIE chromaticity coordinates of the platelets varying from blue to cold white to the warm white region depending on the type of organoamine used in their synthesis.

**Figure** **2**a displays the X‐ray diffraction patterns collected from the RPLP fabricated with the three different organoamine types. The patterns show one family of equally spaced sharp (0 0 2*l*) diffraction peaks corresponding to a *d‐*spacing (periodicity of the inorganic/organic bilayer) that decreases from type I (25.72 ± 0.09 Å) to type II (22.21 ± 0.12 Å) to type III (21.29 ± 0.11 Å), see Figure S3a, Supporting Information. Considering 6 Å for a single [PbBr_4_]^2−^ octahedra layer, the thickness of the dielectric barrier provided by the pair of organoammonium molecules is therefore 19.72, 16.21, and 15.29 Å, respectively. These values of organic interlayer thickness are much lower than what could be expected from fully stretched organoammonium cations. This indicates that there is a strongly reduced van der Waals gap between layers of organoammonium cations that can be fully interpenetrated when changing from type I to type III cations, and/or an increase of the tilting of the cations. Scanning electron microscopy (SEM) images acquired from the dried crystals (Figure S4, Supporting Information) show a characteristic platelet‐like morphology with lateral sizes between 5 to 50 µm. Chemical analysis conducted via μ‐X‐ray fluorescence confirms a Pb:Br ratio of 1:4 for all the produced crystals (Figure S5, Supporting Information), as expected for the formation of RPLP with *n*  =  1.

Under daylight the different deposits of platelets appear with slightly different grey tones. Intriguingly, under UV light they show remarkably different colors: blue for type I, yellow/orange for type II, and white for type III (Figure 2b), which stem from the different profiles of their emission spectra (Figure 2c). The PL profile of the type I sample is typical for RPLP from this amine family^[^ ^12^, ^30^ ^]^ and manifests a narrow emission peak at 406 nm (3.05 eV), with a shoulder around 395 nm (3.14 eV) and a long tail that spans up to 700 nm (1.77 eV). The platelets synthesized with organoamines containing a methyl group (type II and type III) show a broadband emission at room temperature, in addition to the blue emission peak, which extends across the entire visible region with an FWHM that reaches almost 0.8 eV. However, the broadband emission of the type II is centered more toward the red spectral region (at 590 nm) compared to type III (at 525 nm), and it has a relatively weaker PL peak in the blue (at 412 nm [3.01 eV]). Instead, the type III crystals show a blue PL peak at 425 nm (2.92 eV) with nearly equal amplitude as the broad emission. Interestingly, we find that the above described PL characteristics can be transferred to samples made by mixing organoamines of different types in their synthesis (see Experimental Section). For example, by mixing type I and type II, or type I and type III, the resulting deposits display both the blue emission peak from type I component and the broadband emission from type II or type III (Figure S6, Supporting Information).

In the absorption spectra displayed in Figure 2d, we observe a clear band edge at wavelengths below 430 nm (Figure 2d) for all samples, and additional peaks at higher energies that originate from confined excitons in the inorganic layers. Here the excitonic band edge peak is at 395 nm for the type I and type II samples, and at 406 nm for the type III. The higher energy excitonic peaks marked in Figure 2d show a similar trend. Both the band edge excitonic peak in absorption (Figure 2d) and the band edge emission (blue peak in Figure 2c) from the type III sample are redshifted with respect to type I and type II. Since the band gap for all samples is clearly above 2.9 eV, the broadband emission cannot result from free excitons.^[^ ^24^ ^]^ Table S5, Supporting Information, summarizes the optical properties of the synthesized crystals, including the calculated band gap, *E*_g_, as detailed in the Experimental Section and reported in Figure S7, Supporting Information.

Figure 2e reports time‐resolved PL data from the three types of synthesized RPLP platelets, measured from platelet ensembles at ambient temperature. The blue and broadband emission peaks have very similar PL decay traces (Figure S8, Supporting Information), which points to a strong coupling of the related recombination channels.^[^ ^12^, ^16^, ^23^, ^29^ ^]^ Furthermore, the PL decay traces related to the broad emission do not depend on the probe wavelength, as demonstrated in Figure S9, Supporting Information. We note that three exponential time decay functions are required to fit the profiles of the type II and type III samples, while only two are needed for type I, reflecting the more complex recombination dynamics of the type II and type III samples. The complete set of PL decay fitting parameters is reported in Table S6, Supporting Information. For the type I platelets we observe a relatively fast decay that results in an average lifetime of around 3.0 ns. A different behavior is seen in type II and type III crystals that have the additional methyl head group in common, and which show a markedly slower decay. However, the decay trace of the type III crystals has a more non‐linear slope, thus a more pronounced difference between faster and slower decay channels. Here we estimated average lifetimes of 16–18 ns for type II, and around 37–39 ns for the type III (Table S6, Supporting Information). Since the PL quantum efficiency (PLQE) (of 2–3%) is roughly the same for the samples with the different organoammonium head groups discussed here, the non‐radiative decay rate is smallest for the type III samples (Table S7, Supporting Information) that have the longest average life time. Temperature‐dependent PL experiments from 5 to 300 K on a type III sample (Figure S10, Supporting Information) show that the absolute and relative intensities of the blue and broad emission peaks vary with temperature, and that the maximum of the broad emission undergoes spectral shifts, as has been reported for similar 2D layered systems .^[^ ^12^, ^15^, ^17^, ^39^ ^]^ Such behavior was attributed to thermal coupling of self‐trapped exciton states (responsible for the broad emission) to free excitons (that lead to blue band edge emission), and depends on the energetic barriers for self‐trapping and detrapping of the excitons. Figure S10, Supporting Information, shows that the intensity of the broad band emission first increases with decreasing temperature from 300 to 225 K, and then toward lower temperatures it decreases while the blue band edge emission strongly increases. The latter indicates reduced exciton trapping at cryogenic temperatures, which should be related to an energy barrier for self‐trapped exciton (STE) formation that exceeds the thermal energy at cryogenic temperatures.

The modifications of the head group are clearly reflected on the Commission Internationale de l'Eclairage (CIE) coordinates of the emission, as can be seen in Figure 2f. The chromaticity coordinates change from cold (type I) to warm (type II) white light, whereas the type III crystals feature coordinates close to the pure white light (0.33, 0.33). The corresponding correlated color temperature (CCT) of the type II and type III crystals, both showing broadband emission, changes from 3538 to 6473 K, accordingly. All types have a CRI above 86 (see Table S5, Supporting Information).

We now elaborate on the underlying emission mechanisms in the different types of structures. The blue emission peak (between 400‐440 nm) with a relatively narrow profile is observed from all the samples and, considering the band gap derived from the absorption spectra, it can be related to recombination of free excitons in the inorganic layer. Its double peak structure results from contributions from self‐absorption within the crystal, and from in‐plane and out‐of‐plane dipole moments that arise due to the different orientations of the crystals in the deposits.^[^ ^30^, ^31^ ^]^ Concerning the broadband emission that we observed from the (100)‐oriented platelets, there is an on‐going discussion on whether this is related to compositional defects (e.g., halide or organic vacancies)^[^ ^40^, ^41^ ^]^ or to STEs,^[^ ^16^, ^23^, ^25^, ^39^ ^]^ which are generated through strong out‐of‐plane lattice deformation in corrugated RPLP structures and Dion‐Jacobson ones.^[^ ^42^ ^]^ The possible influence of structural defects on the emission can be evaluated by a combined analysis of excitation‐power‐dependent PL measurements and XPS. The results are displayed in Figures S11 and S2, Supporting Information, respectively. Both type II and type III RPLP show a linear dependence on the excited power density in the range of 1–180 mW cm^−2^, with power‐law fitting constant *k* for the broadband emission close to 1, and therefore this data set does not indicate a defect‐assisted radiative recombination.^[^ ^40^, ^41^ ^]^ Concerning the elemental surface quantitative XPS analysis (Table S2, Supporting Information), the type II sample shows a stoichiometry close to the expected one, with a Pb:Br atomic ratio of 1:4, while there is an excess of Pb on the surface of the type III samples. The slightly reduced organic cation content and the excess of Pb in type III crystals could indicate the potential presence of vacancies on the surfaces of the sample. However, the broadband emission is preserved also after increasing the organic cation and Br content in the synthesis (see Figure S12, Supporting Information). Therefore, we conclude that organic and halide vacancies are not at the origin of the observed broad emissions, which are more consistently explained by our structural and optical data as originating from STE.

**Figure 3:**
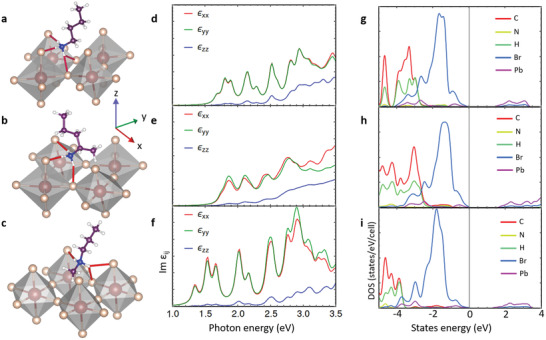
Theoretical analysis of organic cations with different bonding group at the anchor point on RPLPs. a–c) Relaxed type I structure calculated from ref. ^[^ ^12^ ^]^ in (a) and modified to obtain type II (b) and type III (c) configurations. Type I organoammonium makes two and one shared NH—Br bonds, type II three NH—Br bonds, and type III results in one plus a shared NH—Br bond. d–f) The corresponding imaginary part of the dielectric permittivity, Im *ε_ij_*, for the incident light linearly polarized along *x* (in red), *y* (green), and *z* (blue) directions, computed using QE code and a 12 × 12 × 1 *k*‐point grid. g–i) Density of states (DOS) derived from the electronic structure, highlighting the contributions of the different atomic species for the RPLP with the different types of cations.

To gain insight into how molecules with different bonding group can possibly occupy the anchor site in RPLP, we used density functional theory (DFT) calculations. To reduce the computational cost, we initially used butylammonium (BA) as a cation (type I molecule with a short aliphatic chain of 4 carbons), for which the refined unit cell of the RPLP structure is reported in literature.^[^ ^12^ ^]^ This structure features two direct H‐bonds to the apical Br ions, and one that is shared between equatorial ones (**Figure** **3**a; see also Movie S1_Type I, Supporting Information). We then modified the bonding group of the molecule and inserted those of the type II and type III organoamines. Cell and geometry relaxation were performed using the PBE exchange‐correlation functional and with spin‐orbit coupling included (see Experimental Section). The relaxation of both type II and type III structures leads to a tensile strain in the *ab*‐plane to accommodate the bulkier “head” of the organoammonium cation. This reduces the energy increase due to steric hindrance while allowing the formation of the NH—Br bonds. For the type II structure (see Movie S2_Type II, Supporting Information), three NH—Br bonds are formed, but at the expense of large steric hindrance due to the addition of the methyl group. In the case of the type III amine (see Movie S3_Type III, Supporting Information), the methyl group points to the center of the void between the octahedra to minimize steric hindrance, slightly displacing the N ion in order to keep the length of the H‐bonds fixed to their minimum energy value.

Here one direct NH bond is formed with an apical Br ion, and another shared one between apical and equatorial Br ions (Figure 3c). Therefore, the type of organoammonium cation has a significant impact on the distortions of the octahedral lattice. The distortions can be quantified by using the octahedral quadratic elongation parameter (λ_oct_) that can be obtained as

(1) $$\begin{array}{c} \lambda_{\text{oct}}=\frac{1}{6}\overset{6}{\underset{i=1}{\sum }}{\left(\frac{l_{i}}{l_{0}}\right)}^{2} \end{array}$$

where *l*_0_ and *l_i_* are the distances between Pb and neighboring Br ions in the bulk structure and in the 2D compounds, respectively. The resulting values of λ_oct_ are reported in Table S8, Supporting Information, together with the Pb–Br–Pb angles. We found that for the type I amine the formation of three H‐bonds already results in a distortion, and the distortion is increased for the type II amines by the presence of the methyl group that is not in line with the rest of the chain and has to be accommodated in the octahedral void. This leads to an elongation of the Pb—Br bonds, and the calculated *ab* cell surface increases accordingly (Table S9, Supporting Information) with aliphatic chains that are much more tilted compared to type I and type III. Here, the in‐plane angles become wider (161°) than in type I (151°), thus creating more space at the anchor site. For the type III amines, the distortion parameter is smaller than 1, thus overall Pb—Br bond lengths decrease and the octahedra contract with almost no tilting of the in‐plane angles. The band structures are reported in Figure S13, Supporting Information, supporting the results in the DOS and the evaluation of epsilon. It is clearly visible how the strong distortion of the octahedra taking place in the type II compound generates a splitting of the bands.

The distortions of the Pb—Br octahedra described above are known to widen the band gap,^[^ ^43^, ^44^ ^]^ thereby affecting the band structure and optical properties of the materials. Grote et al.^[^ ^44^ ^]^ suggested that this effect is related to the lower Pb—Br overlap upon distortion, which decreases the energy of the valence band more strongly than that of the conduction band. Furthermore, Figure S13, Supporting Information, shows that stronger distortions as in the type II structures lead to an increased splitting of the energy levels. To model the absorbance of these structures, we calculate the imaginary part of the dielectric permittivity (Im ε), as described in ref. ^[^ ^31^ ^]^. Shifts of the absorption edge to lower energies from type I to type II to type III (Figure 3d–f) are in good agreement with the absorbance measurements reported in Figure 2d. The ε_zz_ component is related to the confined dimension, with the dipole along the crystallographic *c*‐axis, and therefore appears at a much higher energy compared to the in‐plane components. This spatial diversity is at the origin of the double peaks in the PL spectra around 400 nm that is observed from deposits with RPLP platelets in different orientations, in line with our previous reports on the type I systems.^[^ ^30^, ^31^ ^]^

### 2.2 Optimizing Emission Efficiency by Tuning the Organoammonium Chain Length

For type III structures we experimentally explored a set of amines with 4 carbons up to 18 carbons in their aliphatic chain. As mentioned before, type III amines with less than 10 carbons did not promote the formation of crystals, not even after long time of incubation. This suggests that the ratio between the lengths of the two components (methyl group vs aliphatic chain) in the type III amines is fundamental to facilitate their accommodation at the anchor point. Therefore, we can infer that a minimum chain length is needed to provide sufficient structural robustness for crystal formation. Also, excess of long alkyl chains could not provide sufficient rigidity to build the RPLP stacking configuration. Here strong chain–chain steric interactions could leave these molecules in an unfavorable configuration/orientation. We tested this hypothesis by using an organoammonium cation made of two long aliphatic chains (*N*‐didodecylamine) in our synthesis and confirmed that such molecules do not favor the crystallization of RPLP structures.

**Figure 4:**
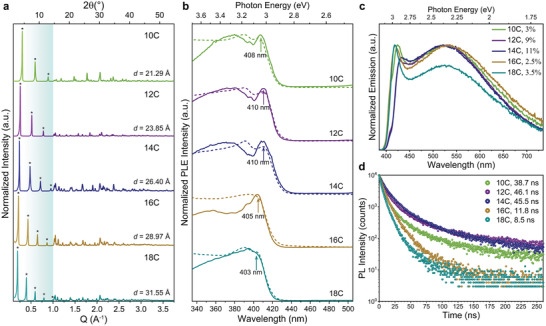
Role of the chain length on the structural and optical properties of type III RPLPs. a) Collection of XRD patterns acquired from the grounded crystals made with type III organoammonium of different aliphatic chain lengths: 10 C atoms in N‐MDA, 12 C in (N‐MDDA), 14 C in (N‐MTDA), 16 C in (N‐MHDA), and 18 C in (N‐MODA). The periodicity of the diffraction peaks (marked with asterisks) decreases with the length of the molecules located between single Pb–Br slabs. b) PLE spectra (solid line) collected at the maxima of the broadband emission compared with the absorption spectra (dashed line) for the studied type III RPLP. c) Emission spectra collected from all the samples. d) PL decay lifetime curves acquired at the maxima of broadband emission for the samples.

The type III amines with more than 10 C atoms in their aliphatic chain lead to micro‐sized crystals similar to the (N‐MDA)_2_PbBr_4_ ones discussed so far (Figure S4, Supporting Information). These are produced with *N*‐methyldodecylammonium (*N*‐MDDA, 12 C), *N*‐methyltetradecylammonium (*N*‐MTDA, 14 C), *N*‐methylhexadecylammonium (*N*‐MHDA, 16 C), and *N*‐methyl‐octadecylammonium (*N*‐MODA, 18 C). **Figure** **4**a displays a collection of XRD patterns of type III samples with different aliphatic chain length (10–18C) from which a linear correlation between the *d‐*spacing and the length of the organoammonium cations is observed (Figure S3b, Supporting Information). Chemical analysis performed via μ‐X‐ray fluorescence spectroscopy confirms a Pb:Br ratio of 1:4 for all the samples prepared with >10C amines (see Figure S5, Supporting Information) and therefore, the chemical composition of the platelets is (*N*‐MDDA)_2_PbBr_4_, (*N*‐MTDA)_2_PbBr_4_, (*N*‐MHDA)_2_PbBr_4_, and (*N*‐MODA)_2_PbBr_4_ made of single octahedra layers (n  =  1). Figure 4b,c displays the absorption, PL excitation (PLE), and PL spectra collected from this set of samples. All of them show identical features regardless of variations in the organoammonium chain length: an absorption band edge in the deep‐blue region, the excitonic absorption peak in the 403–408 nm range (Figure 4b), and PL spectra with a sharp blue peak around 400–430 nm and a broad emission profile in the visible region centered around 525 nm (Figure 4c). Remarkably, all the RPLP structures synthesized with type III molecules show pure white light chromaticity coordinates (Figure 2f) and the corresponding CCT are in the range from 6000–7000 K with CRI values of around 90 (Table S5, Supporting Information).

The PLE spectra with detection at the center of the broadband emission (full lines in Figure 4b) resemble the absorption spectra, and confirm that there are no electronic states below the band edge, and additionally exclude contributions from other sample populations. PLE spectra for different detection wavelengths are shown in Figure S14, Supporting Information, and are almost identical, which excludes also sample heterogeneity. Similar behavior has been observed in, for example, halide double perovskites and other RPLP whose broad emission has been attributed to solely lattice distortions.^[^ ^16^, ^19^, ^23^ ^]^

Interestingly, the PL decay traces and the PLQE are strongly influenced by the aliphatic chain length. Increasing the chain length from 10 to 14C reduces the weight from the fast decay component (that is in the range from 5–9 ns) to the slower one (range from 20–30 ns), and therefore increases their average lifetime from 36 to 45 ns. This correlates with a marked increase in PLQE from 3% to 11% (see Figure S15, Supporting Information), which is competitive with values reported for white emitting RPLP corrugated crystals, around 9%.^[^ ^23^ ^]^ For aliphatic chains that are longer, 16C and 18C, we observe drastically shorter lifetimes (around 4 and 10 ns) and a strongly reduced PLQE of 3%, which points to a markedly increased non‐radiative recombination for such longer chains systems (Table S7, Supporting Information). From our power‐dependent PL measurements on the platelets prepared with the shortest type III molecules (10C), and those that display the highest PLQE (14C), we obtain a *k*‐value closer to 1 from the 14C samples (Figure S11, Supporting Information), indicating that defects are not participating in their radiative recombination mechanism.^[^ ^40^ ^]^ Accordingly, the broadband emission is preserved when passivating the platelets with additional Br/amine treatment (Figure S12, Supporting Information).

One possible reason for the observed dependence of the PLQE on the organoammonium chain length is that the strong interaction between long aliphatic chains affects the degrees of freedom of the anchoring head group and more cation bending can occur when using longer chains, preventing the binding of neighboring molecules. This could significantly affect the surface coverage, as it has been demonstrated, for example, in the assembly of thiol molecules on gold surface.^[^ ^45^ ^]^ In our case, our observations point toward two competitive driving forces: i) van der Waals interactions that can be maximized with longer molecules and ii) steric hindrance caused by the movements of the chains during the synthesis, which increases with their degrees of freedom (number of possible molecule rotations rises with the number of atoms in the chain).^[^ ^46^ ^]^ Within this line of reasoning, the surface coverage likely gets reduced (presence of “pinholes”) when synthesizing RPLP with organic molecules with more than 14C due to their stronger ability to tilt and bend. Molecular bending is clearly visible in the 12C structure for the initial C segments of the aliphatic long chain (Figure S16, Supporting Information), solved and refined from synchrotron single crystal X‐ray diffraction data collected at 100 K (see details in Figures S16,S17 and Tables S3,S4,S10, Supporting Information). Therefore, the aliphatic chain length has a strong impact on the stability of the structure and defect passivation. For the emission efficiency there is a sweet spot in the aliphatic chain length around 12–14 carbons, which could be related either to steric hindrance during the growth process, or to the rigidity of the organic layer that results in a compromise of structural stability and rotational and bending flexibility of the head group for the passivation of the Br ions.

As a final point, from the single crystal XRD refinement, we found that the organoammonium cations in MDDA are well‐aligned and perpendicular to the inorganic layer (see Figure S16, Supporting Information), which is further confirmed by the linear dependence of the *d*‐spacing with respect to the increase of 2C in the aliphatic chain (see Figure S4b, Supporting Information). To explore the effect of the length of the organic cation on the electronic structure, we compared the band structure and the density of states of the experimental (*N*‐MDDA)_2_PbBr_4_ structure (12C) and of an artificial structure with a shortened cation (4C) with the same bonding group (type III). We found that the dispersion of the bands near the gap is not strongly affected, while the band gap is increased by 0.5 eV in the artificial 4C structure (Figure S18, Supporting Information). Therefore shorter‐chain systems may serve as an adequate model that allows to address the effect of the head group on the optical properties at a lower computational cost, as the matching between the experimental and the simulated H‐binding geometry shows (Figure S19, Supporting Information), but it is of limited use for describing the full complexity of the properties since interactions between longer chains can give rise to different distortions.

In summary, we have synthesized a new set of single‐layer RPLP platelets with tunable color coordinates by carefully selecting different architectures of organoammonium molecules that allowed us to systematically compare the structural and optical properties of the resulting structures. By modifying the anchoring group and chain length of the organic cations we obtained broadband light emission that covers the full visible spectral range. The careful tailoring of the cations enabled tuning of the chromaticity of the emission, from cold blue to warm white, and pure white colors, with best values of 11% PLQE, CRI of 90, and color temperature close to 7000 K, and well‐equilibrated systems can provide efficient white emitting materials. This work offers a robust guideline to design, control, and enhance properties in 2D layered perovskites.

## 3. Experimental Section

### Materials

PbBr_2_ (98%), hydrobromic acid (HBr, 48% m/m in water), *N*‐butylmethylamine (96%), *N*‐methylpentylamine (98%), *N*‐hexylmethylamine (96%), *N*‐methyl‐*N*‐octylamine, 1‐methyldecylamine, *N*‐methyldodecylamine (97%), *N*‐methylhexadecylamine‐hydrochloride, *N*‐methyloctadecylamine (98%), undecylamine, and didodecylamine (≥97%) were purchased from Sigma‐Aldrich. *N*‐methyldecylamine (95%) and *N*‐methyltetradecylamine (95%) were purchased from AKos GmbH. All the chemicals were used without further purification.

### Synthesis

Stoichiometric amounts of PbBr_2_ (95.5 mg, 0.26 mmol) and HBr (60 µL, 0.52 mmol) were added to 1 mL of acetone forming a clear and transparent solution after vigorously shaking the mixture. Then, the selected amine (0.62 mmol) was injected into the mixture. In the case of the *N*‐methyltetradecylamine, *N*‐methylhexadecylamine, and *N*‐methyloctadecylamine, the powders were dissolved in acetone by sonication for a minute prior to injection. The crystals were formed within few minutes under strong magnetic stirring and collected for purification. This step was performed by first washing the crystals with HBr followed by four times in acetone to remove the unreacted precursors. Finally, the crystals were dried overnight on filter paper. In the case of the crystals prepared with type I‐type II and type I‐type III, 0.3 mmol of each amine were first mixed in a vial and then added to the transparent solution made with PbBr_2_ and HBr in acetone, as described above. The powders were centrifuged at 5500 RPM for 5 min and dried in a filter paper for the PL analysis. Post‐treatment on (*N*‐MTDA)_2_PbBr_4_ crystals were carried out by immersing the crystals overnight in 50 mg mL^−1^ *N*‐MTDABr solution in methanol. 1.2 mmol of *N*‐methyltetradecylamine (273 mg) and HBr (136 µL) were added in the synthesis to achieve more *N*‐MTDA and Br sources. Similar post‐treatment was performed on the type III‐C10 and C12 crystals. All the experiments were conducted at room temperature. Note that all the characterizations were performed on dried samples. However, the crystals could be dispersed in acetone, toluene, ethanol, and methanol (Figure S20, Supporting Information) without quenching of their emission.

### Structural and Morphological Characterization

Scanning electron microscopy analysis on the resulting 2D perovskites was performed on an FEI Nova 600 NanoLab instrument by depositing the dried crystals directly on Si substrates. X‐ray diffraction studies were conducted on a Rigaku SmartLab system, equipped with a 9 kW rotating Cu anode working at 40 kV and 150 mA and a D/teX Ultra 1D silicon strip detector on finely grounded samples, prepared by using an agate mortar and pestle and deposited on zero‐diffraction Si substrates. Synchrotron single crystal diffraction. Single‐crystal X‐ray diffraction data measurements for the platelets made of (N‐MDDA)_2_PbBr_4_ were carried out at the beamline PXIII (X06DA‐PXIII) at the Swiss Light Source (SLS), Villigen, Switzerland. The measurements were collected using a Parallel Robotics Inspired (PRIGo) multi‐axis goniometer^[^ ^47^ ^]^ and a PILATUS 2M‐F detector. Data collection was performed at low temperature (*T* = 100 K) on a selected crystal mounted on litholoops (Molecular Dimensions). Complete data were obtained by merging two 360° ω scans at χ = 0° and χ = 30° of PRIGo. In shutterless mode, a 360° data set was collected in 3 min (beam energy of 17 keV, λ= 0.72931 Å, focus size 90 × 50 µm^2^, 0.25 s of exposure time per frame, 0.5° scan angle).

Main experimental details are given in Table S8, Supporting Information; additional tables, concerning refined fractional atomic coordinates, displacement parameters, bond distances and angles, torsion angles, and hydrogen bonds, are provided in the Supporting Information (Tables S9,S10, Supporting Information, and CIF file; structure named: N‐MDDA‐100K).

Diffraction data were analyzed by XDS,^[^ ^48^ ^]^ a software consisting of eight subroutines able to carry out the main data reduction steps; the process of scaling and correction for absorption effects of the integrated intensities was performed by the XSCALE subroutine.^[^ ^48^ ^]^ Structure solution was carried out by Direct Methods^[^ ^49^ ^]^ using SIR2019;^[^ ^50^ ^]^ the partial structure model provided by SIR2019 was completed and refined by SHELXL2014/7.^[^ ^51^ ^]^ All non‐hydrogen atoms were refined anisotropically; the H atoms were positioned via electron density map calculated by difference Fourier synthesis and their fractional coordinates were freely refined. The programs used to prepare material for publication were WinGX^[^ ^52^ ^]^ and publCIF;^[^ ^53^ ^]^ the software Mercury^[^ ^54^ ^]^was applied for molecular graphics.

### X‐Ray Photoelectron Spectroscopy

The UDA, 1‐MDA, and *N*‐MDA samples were prepared by pressing a few milligrams of finely ground RPLP platelets powder onto high purity indium pellets (Sigma‐Aldrich). Measurements were carried out using a Kratos Axis Ultra^DLD^ spectrometer (Kratos Analytical Ltd., UK) using a monochromatic Al Kα source (hν = 1486.6 eV) operated at 20 mA and 15 kV. The analyses were performed on 300 × 700 µm^2^ area. High‐resolution spectra were collected at a pass‐energy of 10 eV and an energy step of 0.1 eV. Data analysis was performed with CasaXPS software (Casa Software, Ltd., version 2.3.22).

### Elemental Analysis

Micro X‐ray fluorescence analyses were performed using a Bruker M4 Tornado µ‐XRF spectrometer equipped with an air‐cooled rhodium tube operated at 50 kV and 200 µA. A silicon drift detector with a 30‐mm^2^ sensitive area and energy resolution of <145 eV for Mn Kα was used. The beam was focused to a spot size of ≈25 µm by using poly‐capillary optics. The measurements were carried out directly on the samples previously placed on the µ‐XRF platform under 20 mbar vacuum conditions. The samples were scanned “on the fly” using a measurement time of 100 ms per pixel with the spacing of 11 µm, by moving the sample stage continuously to create the 2D map. Spectra acquisition, elemental map evaluation, and quantification were done based on the fundamental parameter method using the Esprit software from Bruker. The reported values were the average of five different measurements with standard deviation below 0.1.

### Optical Characterization

The absorption spectra were collected from the dried crystals by using a Varian Cary 5000 ultraviolet–visible–near infrared (UV–vis–NIR) spectrophotometer equipped with an external diffuse reflectance accessory, and operating in absorption geometry. To estimate the bandgap (*E*_g_) of the materials, Tauc plots were calculated from the diffused reflectance spectra using the Kubelka–Munk equation. *E*_g_ was estimated by extrapolating the absorption edge to the imaginary line parallel to the energy axis where the absorption edge was interrupted by the excitonic absorption.^[^ ^17^ ^]^ The PL, PLE spectra, power‐dependent PL, and PLQE measurements were conducted on an Edinburgh Instruments (FLS920) fluorescence spectrometer equipped with a Xenon lamp with monochromator for steady‐state PL excitation. The PL spectra were collected with an excitation wavelength of 375 nm. The PL and PLE spectra were collected with 1 nm resolution and 0.5 s dwell time. The PLQE values were obtained from five different samples from different synthesis batches by exciting them at 375 nm using a calibrated integrating sphere with step increments of 1 nm and integration time of 0.2 s per data point for five repeats. Light absorption due to scattering inside the sphere was taken into account for the PLQE calculation by collecting three different spectra: 1) directly exciting the sample in the sphere, 2) indirectly exciting sample in the sphere, 3) without the sample in the sphere. Three measurements were performed on each sample of layered perovskite microcrystals with a standard experimental error below 1%. Time resolved PL measurements were carried out with a time correlated single‐photon counting (TCSPC) unit coupled to a pulsed diode laser. The samples were excited at 375 nm with picosecond pulses at a repetition rate of 0.5 MHz and a spectral collection window of 10 nm. The samples for all these studies were prepared by placing the RPLP crystals in between two clean glass substrates. The excitation power density dependent photoluminescence measurement was carried out by using an electronic pulsed diode laser at 375 nm with 50 ns pulse period. The average intensity of the pulse was monitored by a power meter. CIE coordinates, CRI and CCT values were obtained from Color Calculator by OSRAM Sylvania, Inc. Steady‐state PL spectra at different temperatures were recorded with an Edinburgh Instruments fluorescence spectrometer (FLS920) coupled to an optical fiber. The RPLP dried crystals were deposited in between two thin sapphire substrate (TedPella) and placed inside a closed‐cycle helium cryostat (Advanced Research Systems, Inc.) under vacuum. The steady‐state PL was collected by exciting the sample with a 375 nm laser diode at 50 ns repetition period.

### Theoretical Analysis

Geometry and cell relaxation were carried out on all the structures using VASP software,^[^ ^55^ ^]^ with the exception of BA_2_Pb_2_Br_4_, where the cell parameters were constrained to the experimental values.^[^ ^12^ ^]^ The threshold for force convergence was set to 10^−5^ eV, whereas the energy threshold for SCF convergence was 10^−6 ^eV. For evaluation of frequency‐dependent dielectric permittivities, norm‐conserving pseudopotentials were used as they were the only ones supported by the epsilon.x code from the Quantum Espresso software package.^[^ ^56^ ^]^ The band structures of the (N‐MDDA)‐based crystals and artificial type III‐C4 structures were calculated using the SIESTA code.^[^ ^57^ ^]^

## Conflict of Interest

The authors declare no conflict of interest.

## Supporting information

Supporting Information

## Data Availability

Research data are not shared.
