# Supplementary material for: Engineering the Optical Emission and Robustness of Metal‐Halide Layered Perovskites through Ligand Accommodation
Source: Adv Mater. 2021 Mar 1;33(13):2008004. doi: 10.1002/adma.202008004 (PMC11468748; doi:10.1002/adma.202008004)
Supplement: Supplementary file 1 — Supporting Information [file ADMA-33-2008004-s001.pdf]

© 2021 Wiley-VCH GmbH

ADVANCED MATERIALS

Supporting Information

for *Adv. Mater.,* DOI: 10.1002/adma.202008004

Engineering the Optical Emission and Robustness of Metal-Halide Layered Perovskites through Ligand Accommodation

Balaji Dhanabalan, Giulia Biffi, Anna Moliterni, Vincent Olieric, Cinzia Giannini, Gabriele Saleh, Louis Ponet, Mirko Prato, Muhammad Imran, Liberato Manna, Roman Krahne, Sergey Artyukhin,* and Milena P. Arciniegas**

Copyright Wiley-VCH GmbH, 2021.

Supporting Information

Engineering the Optical Emission and Robustness of Metal-Halide Layered Perovskites through Ligand Accommodation

Balaji Dhanabalan,[Δ] *Giulia Biffi,*[Δ] *Anna Moliterni, Vincent Olieric, Cinzia Giannini, Gabriele Saleh, Louis Ponet, Mirko Prato, Muhammad Imran, Liberato Manna, Roman Krahne,* Sergey Artyukhin,* and Milena P. Arciniegas**

Table S1. List of amines studied for the preparation of RPLP structures. The *d*-spacing was calculated from the collected XRD patterns. NC: No crystals are formed under the reported synthesis conditions.

		C atoms aliphatic chain	Molar mass, g/mol	*d*-spacing, Å	Estimated organic layer, Å	Emission Color
**Aliphatic Primary amines**	N-butylamine[1]	4	73.14	13.8	7.8	Deep Blue
	N-octylamine[1]	8	129.24	21.1	15.1	Bluish- White
	N-decylamine[1]	10	157.3	24.1	18.1	Bluish- White
	N-undecylamine	11	171.33	25.7	19.7	Bluish- White
	N-dodecylamine	12	185.35	27.2	21.2	Bluish- White
	1-methyldecylamine	10	171.33	22.2	16.2	Yellowish-White
	1-methyldodecylamine	12	199.38	24.7	18.7	Yellowish-White
**Aromatic primary amines**	N-benzylamine[1]	-	107.15	16.7	10.7	Deep Blue
	N-phenethylamine[1]	-	121.18	16.7	10.7	Deep Blue
	N-dopamine hydrocloride[1]	-	189.64	17.1	11.1	Deep Blue
**Aliphatic Secondary amines**	N-didodecylamine	12	353.67	NC	-	-
	N-methylbutylamine	4	87.16	NC	-	-
	N-methylpentylamine	5	101.70	NC	-	-
	N-methylhexylamine	6	115.22	NC	-	-
	N-methyloctylamine	8	143.27	NC	-	-
	N-methyldecylamine	10	171.33	21.3	15.3	White
	N-methyldodecylamine	12	199.38	23.9	17.85	White
	N-methyltetradecylamine	14	227.43	26.4	20.4	White
	N-methylhexadecylamine hydrochloride	16	291.95	29.0	22.9	White
	N-methyloctadecylamine	18	283.54	31.6	25.5	White
**Aromatic secondary amines**	N-methylbenzylamine	-	121.18	NC	-	-
	N-methylphenethylamine	-	135.21	NC	-	-

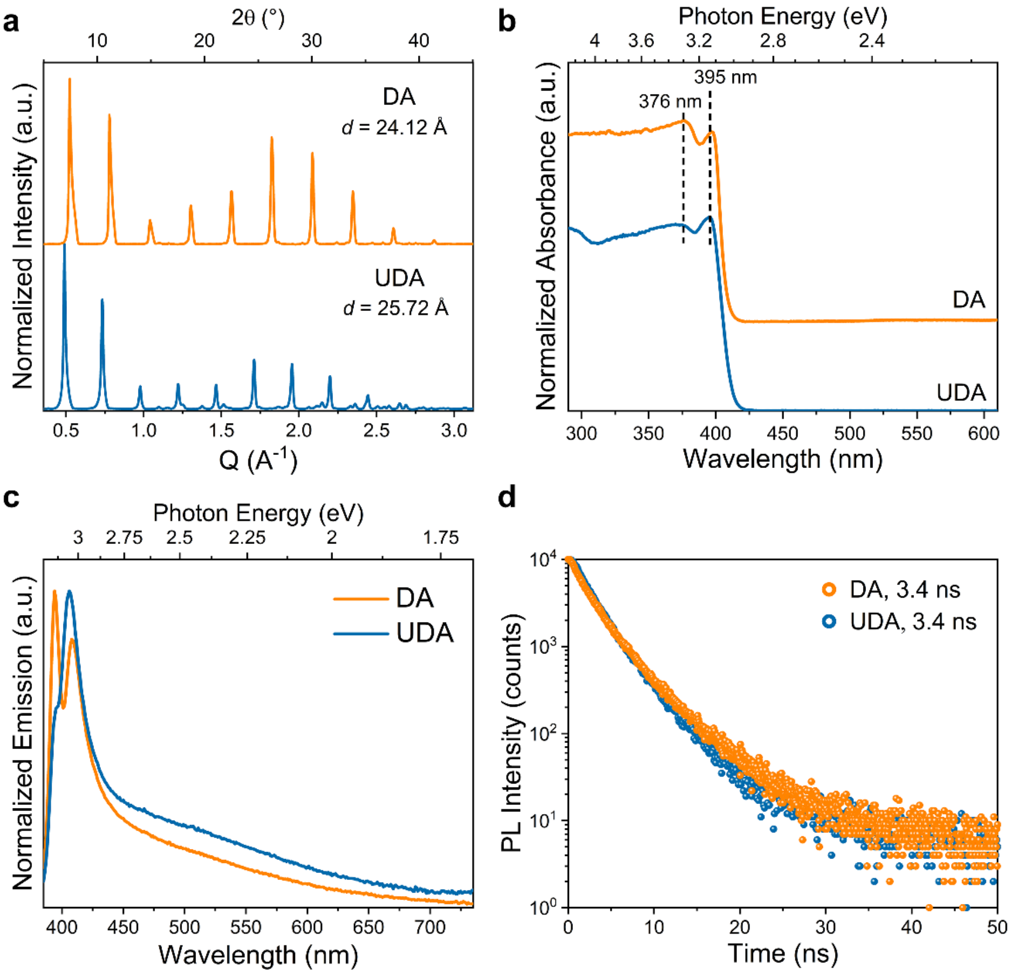

Figure S1. Experimental data collected from RPLP crystals synthesized by two different type I amines, undecylamine (UDA) and decylamine (DA) showing their similar characteristics.

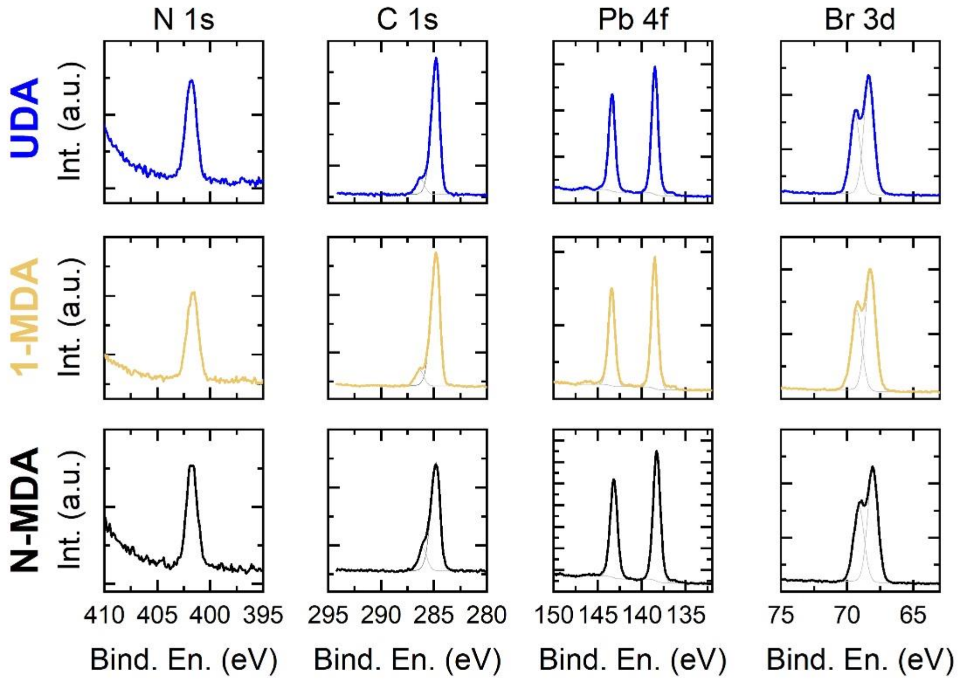

Figure S2. XPS data acquired on the $(UDA)_2PbBr_4$ (top panels), $(1\text{-}MDA)_2PbBr_4$ (middle panels), and $(N\text{-}MDA)_2PbBr_4$ (bottom panels) samples assessing the chemical state of nitrogen, lead and bromine. The data were acquired over the typical energy ranges for N 1s (first column from the left), C 1s (second column), Pb 4f (third column), and Br 3d (fourth column) peaks.

The C 1s spectra can all be fitted with two peaks: i) an intense peak at 284.8 eV, which is associated to the CH_2-CH_2 aliphatic backbone of the used organoammonium cations, and ii) a peak of lower intensity located at 286.3 eV in the UDA and 1-MDA samples, and at 286.0 eV in the N-MDA sample that is related to the C-N bonds. Note that the position and relative intensity of the second peak are similar for UDA and 1-MDA (in both samples there is one C-N bond in the molecule), while in the case of N-MDA the higher intensity is consistent with the presence of two carbons bonding the nitrogen. Also, the position of the peak in this last case is slightly shifted to lower binding energies, reflecting the different chemical environment to some extent.

Table S2. Summary of the binding energy positions of the observed XPS peak (error: ± 0.2 eV) and results of the quantitative analysis. Note that the N:Br atomic ratio results in ca. 2.0, which rules out a partial desorption of both Br and organics under the X-rays during XPS data acquisition.

	**N 1s (eV)**	**C 1s C-C (eV)**	**C 1s C-N (eV)**	**Pb $4f_{7/2}$ (eV)**	**Br $3d_{5/2}$ (eV)**	**N at.%**	**Pb at.%**	**Br at%**	**Surface stoich.**
**$(UDA)_2PbBr_4$**	401.8	284.8	286.3	138.5	68.3	25.6	21.5	52.9	$(UDA)_{1.9}Pb_{1.6}Br_4$
**$(1\text{-}MDA)_2PbBr_4$**	401.7	284.8	286.3	138.5	68.2	27.5	16.8	55.7	$(1\text{-}MDA)_2Pb_{1.2}Br_4$
**$(N\text{-}MDA)_2PbBr_4$**	401.7	284.8	286.0	138.3	68.1	23.3	21.9	54.8	$(N\text{-}MDA)_{1.7}Pb_{1.6}Br_4$

Table S3. Geometric parameters (Å, º) for $(N\text{-}MDDA)_2PbBr_4$ platelets. Data collected at 100 K.

Pb1—Br1	2.9304 (4)	C9—C8	1.522 (5)
Pb1—Br1^{i}	2.9304 (4)	C9—C10	1.527 (5)
Pb1—Br2^{i}	2.9794 (4)	C9—H9A	0.96 (6)
Pb1—Br2	2.9795 (4)	C9—H9B	1.00 (5)
Pb1—Br1^{ii}	3.1561 (4)	C8—H8A	1.02 (5)
Pb1—Br1^{iii}	3.1561 (4)	C8—H8B	1.01 (6)
Br1—Pb1^{iv}	3.1561 (4)	C10—C11	1.524 (5)
C3—C2	1.509 (6)	C10—H10A	1.02 (5)
C3—C4	1.534 (5)	C10—H10B	1.01 (6)
C3—H3A	0.95 (5)	C11—C12	1.523 (5)
C3—H3B	0.99 (5)	C11—H11A	0.99 (6)
C5—C4	1.517 (5)	C11—H11B	1.01 (6)
C5—C6	1.521 (5)	C6—H6A	0.97 (6)
C5—H5A	0.99 (5)	C6—H6B	1.01 (5)
C5—H5B	0.95 (5)	C4—H4A	1.02 (5)
N1—C1	1.480 (6)	C4—H4B	1.01 (5)
N1—C2	1.499 (5)	C1—H1A	0.87 (6)
N1—HN1A	0.99 (6)	C1—H1B	1.07 (7)
N1—HN1B	0.81 (6)	C1—H1C	0.83 (7)
C7—C8	1.523 (5)	C12—C13	1.521 (6)
C7—C6	1.526 (5)	C12—H12A	0.99 (6)
C7—H7A	0.97 (5)	C12—H12B	1.00 (6)
C7—H7B	1.01 (6)	C13—H13A	1.07 (7)
C2—H2A	0.98 (5)	C13—H13B	0.94 (6)
C2—H2B	0.96 (5)	C13—H13C	1.03 (7)
Br1—Pb1—Br1^{i}	96.808 (17)	C10—C9—H9B	107 (3)
Br1—Pb1—Br2^{i}	91.062 (10)	H9A—C9—H9B	113 (4)
Br1^{i}—Pb1—Br2^{i}	88.165 (10)	C9—C8—C7	113.3 (3)
Br1—Pb1—Br2	88.162 (10)	C9—C8—H8A	108 (3)
Br1^{i}—Pb1—Br2	91.058 (11)	C7—C8—H8A	108 (3)
Br2^{i}—Pb1—Br2	178.831 (17)	C9—C8—H8B	107 (3)
Br1—Pb1—Br1^{ii}	94.781 (3)	C7—C8—H8B	109 (3)
Br1^{i}—Pb1—Br1^{ii}	168.397 (16)	H8A—C8—H8B	111 (4)
Br2^{i}—Pb1—Br1^{ii}	91.049 (10)	C11—C10—C9	113.5 (3)
Br2—Pb1—Br1^{ii}	89.888 (10)	C11—C10—H10A	109 (3)

Br1—Pb1—Br1^{iii}	168.397 (16)	C9—C10—H10A	108 (3)
Br1^{i}—Pb1—Br1^{iii}	94.780 (4)	C11—C10—H10B	109 (3)
Br2^{i}—Pb1—Br1^{iii}	89.888 (10)	C9—C10—H10B	106 (3)
Br2—Pb1—Br1^{iii}	91.048 (10)	H10A—C10—H10B	112 (4)
Br1^{ii}—Pb1—Br1^{iii}	73.638 (15)	C12—C11—C10	113.8 (3)
Pb1—Br1—Pb1^{iv}	165.465 (15)	C12—C11—H11A	109 (3)
C2—C3—C4	111.4 (3)	C10—C11—H11A	108 (3)
C2—C3—H3A	105 (3)	C12—C11—H11B	108 (3)
C4—C3—H3A	112 (3)	C10—C11—H11B	107 (3)
C2—C3—H3B	112 (3)	H11A—C11—H11B	110 (4)
C4—C3—H3B	110 (3)	C5—C6—C7	113.6 (3)
H3A—C3—H3B	106 (4)	C5—C6—H6A	109 (3)
C4—C5—C6	112.8 (3)	C7—C6—H6A	107 (3)
C4—C5—H5A	108 (3)	C5—C6—H6B	109 (3)
C6—C5—H5A	110 (3)	C7—C6—H6B	111 (3)
C4—C5—H5B	106 (3)	H6A—C6—H6B	107 (4)
C6—C5—H5B	107 (3)	C5—C4—C3	114.3 (3)
H5A—C5—H5B	112 (5)	C5—C4—H4A	107 (3)
C1—N1—C2	114.6 (3)	C3—C4—H4A	109 (3)
C1—N1—HN1A	107 (3)	C5—C4—H4B	109 (3)
C2—N1—HN1A	109 (3)	C3—C4—H4B	111 (3)
C1—N1—HN1B	110 (4)	H4A—C4—H4B	106 (4)
C2—N1—HN1B	109 (4)	N1—C1—H1A	103 (5)
HN1A—N1—HN1B	106 (4)	N1—C1—H1B	107 (3)
C8—C7—C6	113.5 (3)	H1A—C1—H1B	117 (5)
C8—C7—H7A	109 (3)	N1—C1—H1C	105 (5)
C6—C7—H7A	107 (3)	H1A—C1—H1C	114 (7)
C8—C7—H7B	107 (3)	H1B—C1—H1C	110 (5)
C6—C7—H7B	107 (3)	C13—C12—C11	113.0 (4)
H7A—C7—H7B	113 (5)	C13—C12—H12A	111 (3)
N1—C2—C3	112.2 (3)	C11—C12—H12A	105 (3)
N1—C2—H2A	107 (3)	C13—C12—H12B	109 (3)
C3—C2—H2A	113 (3)	C11—C12—H12B	107 (3)
N1—C2—H2B	106 (3)	H12A—C12—H12B	112 (5)
C3—C2—H2B	108 (3)	C12—C13—H13A	115 (3)
H2A—C2—H2B	110 (4)	C12—C13—H13B	113 (4)
C8—C9—C10	113.4 (3)	H13A—C13—H13B	107 (5)

C8—C9—H9A	108 (3)	C12—C13—H13C	109 (4)
C10—C9—H9A	105 (3)	H13A—C13—H13C	105 (5)
C8—C9—H9B	110 (3)	H13B—C13—H13C	107 (5)
C1—N1—C2—C3	67.4 (5)	C4—C5—C6—C7	-175.2 (4)
C4—C3—C2—N1	164.6 (3)	C8—C7—C6—C5	-176.7 (4)
C10—C9—C8—C7	-176.1 (4)	C6—C5—C4—C3	-178.6 (4)
C6—C7—C8—C9	-176.0 (4)	C2—C3—C4—C5	69.1 (5)
C8—C9—C10—C11	-177.2 (4)	C10—C11—C12—C13	-179.1 (4)
C9—C10—C11—C12	-175.8 (4)		

Table S4. Hydrogen-bond geometry (Å, º) for $(N\text{-}MDDA)_2PbBr_4$ platelets. Data collected at 100 K.

D—H···*A*	*D*—H	H···*A*	*D*···*A*	*D*—H···*A*
C3—H3*B*···Br2^{i}	0.99 (5)	3.08 (5)	3.844 (4)	134 (3)
N1—H*N*1*A*···Br2	0.99 (6)	2.31 (6)	3.265 (4)	162 (4)
N1—H*N*1*B*···Br1	0.81 (6)	3.02 (5)	3.488 (3)	120 (4)
N1—H*N*1*B*···Br1^{ii}	0.81 (6)	2.97 (5)	3.624 (3)	139 (4)
C2—H2*B*···Br2^{iii}	0.96 (5)	2.92 (5)	3.564 (4)	126 (3)

Symmetry codes: (i) *x*+1, *y*, *z*; (ii) -*x*+1, *y*, -*z*+1/2; (iii) -*x*+1/2, *y*-1/2, *z*.

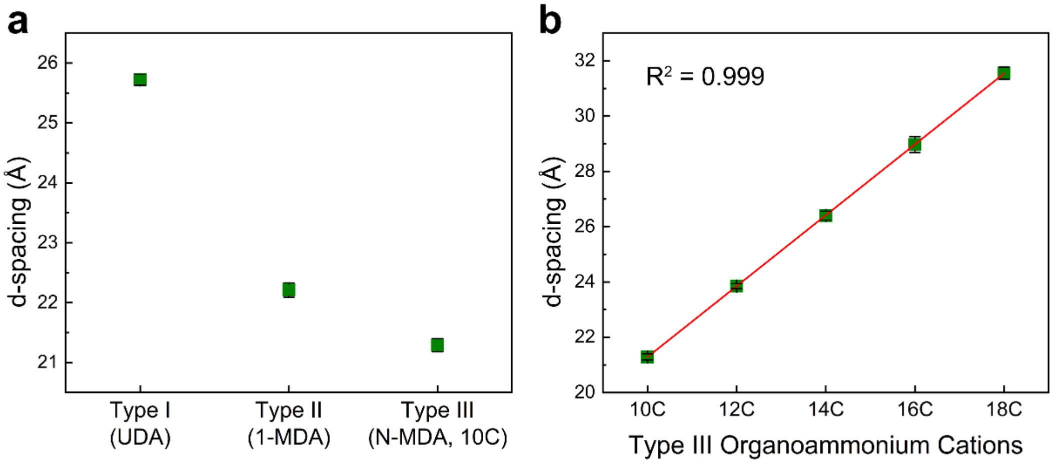

Figure S3. *d*-spacing calculated from the periodic diffraction peaks observed on the XRD patterns as a function of (a) the different types of organoammonium cations of the fabricated RPLPs and (b) the alkyl chain length in the type III molecules. We observe a linear correlation between the organic interlayer spacing and the length of the type III organoammonium cations in (b). The *d*-spacing increases by 2.54 Å for two additional C atoms in the long alkyl chain, which corresponds very well with the length of the 2C segment obtained in our simulations.

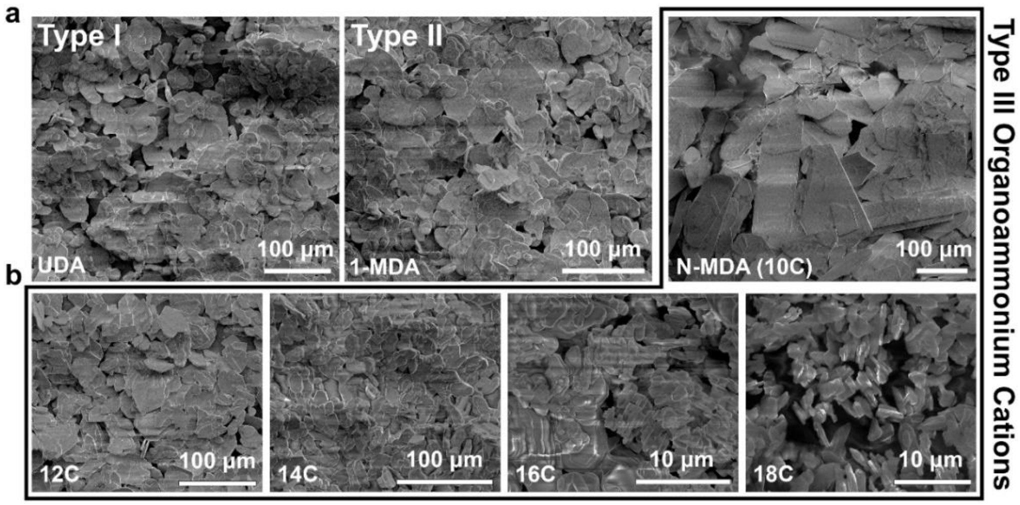

Figure S4. SEM images of the synthesized RPLP when using the type I (undecylamine, UDA), type II (1-methyldecylamine, 1-MDA), and type III (N-MDA (10C), 12C, 14C, 16C, 18C) organoamines in their synthesis. The crystals with comparatively short aliphatic chain display an extended lateral size of about 50 µm (top row) while long molecules produce crystals with dramatically reduced sizes of about 1 to 5 µm (for example, 16C and 18C-based platelets).

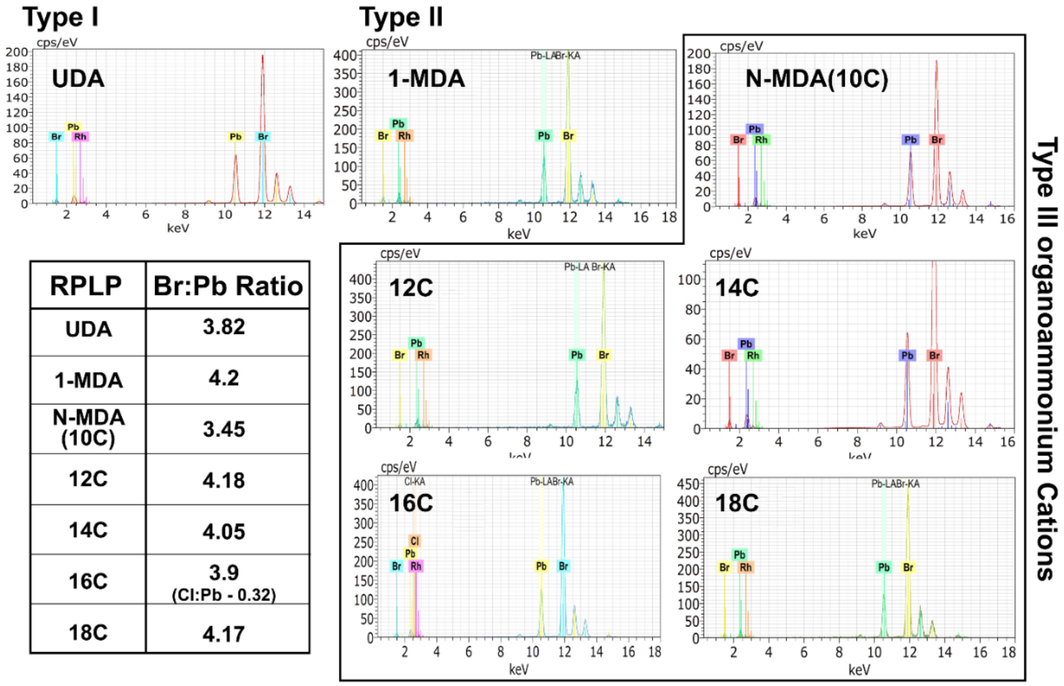

RPLP	Br:Pb Ratio
UDA	3.82
1-MDA	4.2
N-MDA (10C)	3.45
12C	4.18
14C	4.05
16C	3.9 (Cl:Pb - 0.32)
18C	4.17

Figure S5. µ-XRF analysis spectra collected from the resulting platelets synthesized with type I (undecylamine, UDA), type II (1-methyldecylamine, 1-MDA), and type III (N-MDA (10C), 12C, 14C, 16C, 18C) organoamines. All the crystals showed Br:Pb ratio around 4.0, with a strongly reduced value from N-MDA-based crystals. 16C-based crystals have a very low content of Cl due to the presence of hydrochloride in the initial amine.

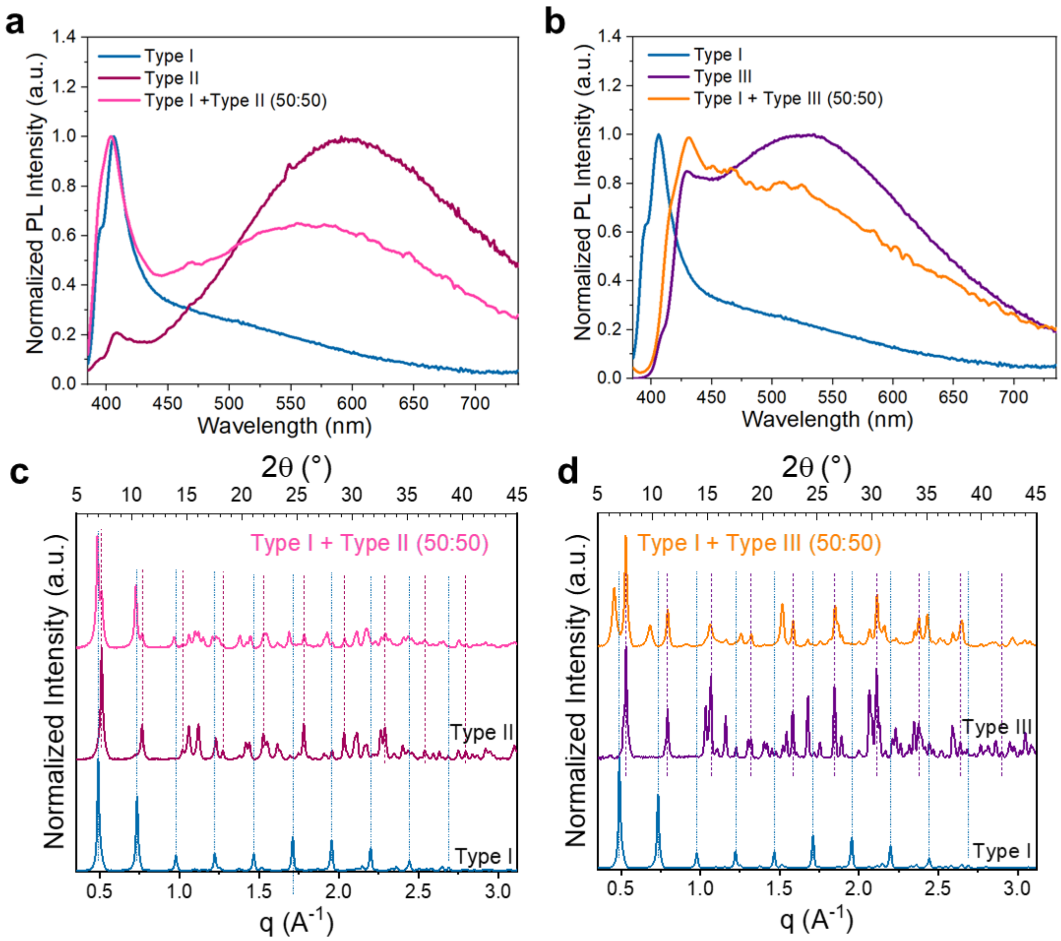

Figure S6. PL and XRD spectra collected from samples made by using a 50:50 mixture of (a, c) type I (UDA) and type II (1-MDDA) and (b, d) type I (UDA) and type III (N-MDDA) organoamines in their synthesis. The spectra of the crystals prepared with the corresponding type of amine is also displayed. The appearance of the double features at small q values in the XRD spectra of the mixtures most likely originates from a superposition of the diffraction patterns from the single types. Therefore, such samples should consist of mixed populations of platelets from both types.

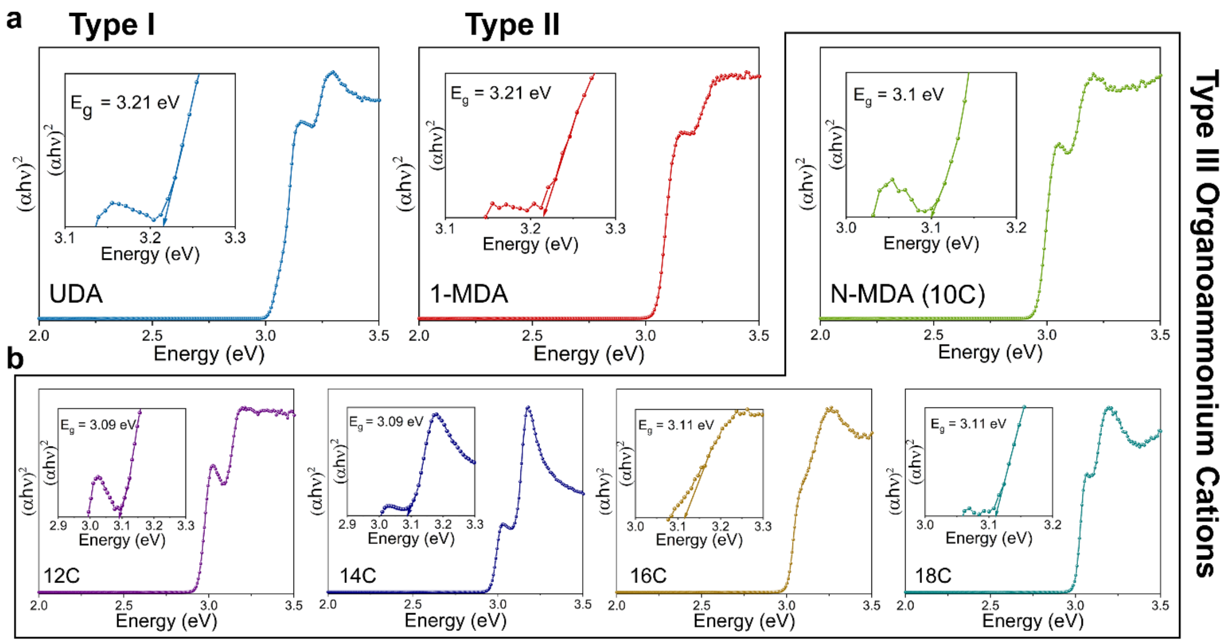

Figure S7. Kubelka-Munk plot of the crystals produced using the (a) three different type of organoamines (b) the alkyl chain length in the type III molecules. Insets show the estimation of bandgap, Eg, by extrapolating the absorption edge to the imaginary line parallel to the energy axis where the absorption edge is interrupted by the excitonic absorption.

The calculated E_g values result in 3.1 eV, 3.09 eV, and 3.11 eV for $(N\text{-}MDA)_2PbBr_4$, $(12C)_2PbBr_4$ and $(14C)_2PbBr_4$, $(16C)_2PbBr_4$ and $(18C)_2PbBr_4$ crystals, respectively. The type I and II crystals show the same Eg value of 3.21 eV, which are slightly higher than those obtained from type III crystals.

Table S5. Optical characteristics at room temperature of the RPLP synthesized using different type of organoamine molecules.

RPLP platelets		E_g, eV	PL Peaks		FWHM, nm (meV)	CIE		CCT, K	CRI	Avg. PLQE, %
			Blue region, nm (eV)	Broadband region, nm (eV)		x	y			
Type I	UDA	3.21	395 (3.14)<br>406 (3.05)	525 (only a long tail)	-	0.22	0.21	-	-	2 ± 0.5
Type II	1-MDA	3.21	412 (3.01)	590 (2.10)	215 (766)	0.42	0.43	3538	86	3 ± 0.5
Type III	N-MDA (10C)	3.10	425 (2.92)	525 (2.36)	196 (879)	0.31	0.35	6473	89	3 ± 0.5
	12C	3.09	430 (2.89)	525 (2.36)	183 (822)	0.30	0.34	6977	87	9 ± 1
	14C	3.09	430 (2.89)	525 (2.36)	186 (809)	0.31	0.35	6700	86	11 ± 1
	16C	3.11	420 (2.95)	530 (2.34)	207 (915)	0.32	0.35	6062	89	2.5 ± 0.5
	18C	3.11	420 (2.95)	530 (2.34)	196 (867)	0.31	0.34	6745	89	3.5 ± 0.5

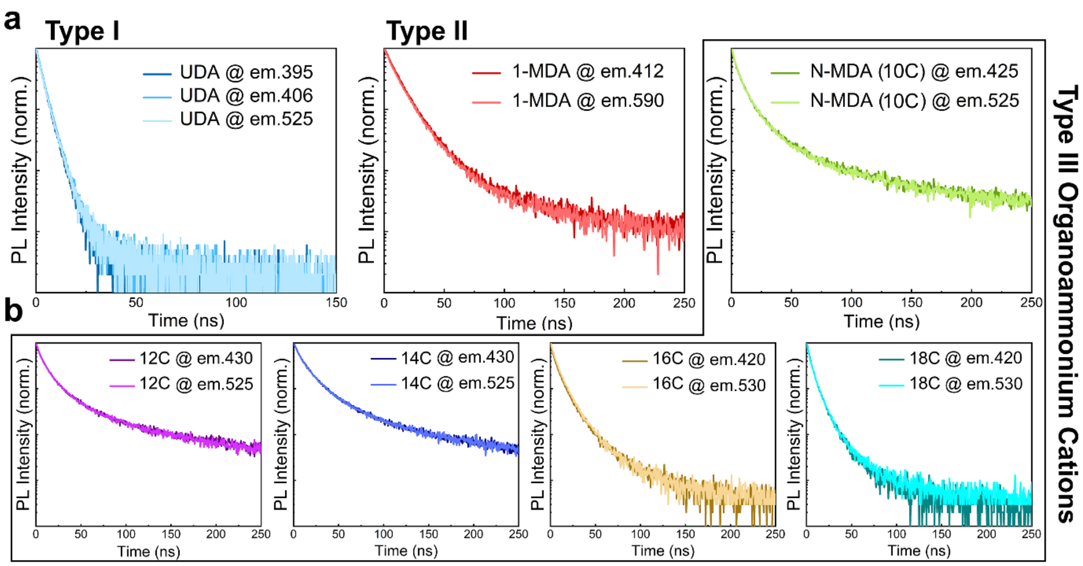

Figure S8. PL decay lifetime profiles collected from the single layer RPLP prepared with the (a) different types of organoamines (b) type III molecules with different alkyl chain length selected in this study. The PL decay traces were acquired at the maxima of the emission peaks in the blue and broadband region reported in Figure 2c of the main manuscript.

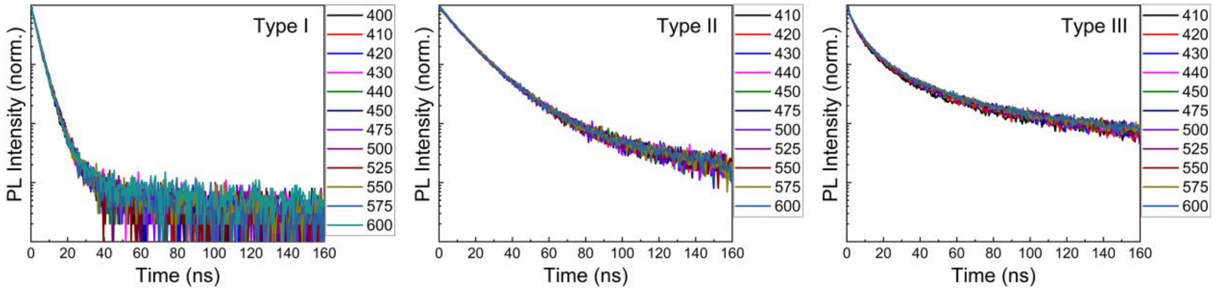

Figure S9. PL decay traces collected at different probe wavelengths from the single layer RPLP prepared with the three types of organoamines. The probe wavelengths are shown in nm in the legends of the panels. The data shows that the probe wavelength does not significantly influence the measured PL decay.

Table S6. Fitting parameters of the PL time decay profiles presented in Figure S5 for all the synthesized crystals by using different types of organoamines. τ_1, τ_2, τ_3, and τ_{avg} are the three-exponential time decay fitting and their average value.

RPLP platelets	PL peak, nm	A_1, %	τ_1, ns	A_2, %	τ_2, ns	A_3, %	τ_3, ns	τ_{avg}, ns
UDA	395	83.9	2.18 ± 0.02	16.1	4.89 ± 0.08	-	-	3.0 ± 0.05
	406	82.1	2.35 ± 0.03	17.9	5.01 ± 0.09	-	-	3.2 ± 0.06
	525	90.1	2.37 ± 0.02	9.9	5.62 ± 0.11	-	-	3.05 ± 0.07
1-MDA	412	19.5	3.79 ± 0.66	77.4	12.95 ± 0.27	3.1	47.54 ± 2.49	16.53 ± 0.8
	590	50.6	7.84 ± 0.63	48.1	16.23 ± 0.96	1.3	71.67 ± 1.47	17.56 ± 0.84
N-MDA (10C)	425	79.3	5.35 ± 0.08	19.5	21.14 ± 0.44	1.2	151.26 ± 2.1	37.16 ± 0.55
	525	81	5.81 ± 0.07	17.79	23.23 ± 0.38	1.21	155.51 ± 1.8	38.70 ± 0.47
12C	430	72.9	6.85 ± 0.1	25.1	26.08 ± 0.39	2	154.12 ± 2.59	46.12 ± 0.81
	525	69.8	6.62 ± 0.11	28.2	24.99 ± 0.38	2	154.66 ± 2.47	46.15 ± 0.73
14C	430	72.6	8.78 ± 0.12	25.6	30.12 ± 0.37	1.8	161.15 ± 2.48	45.19 ± 0.69
	525	72.7	8.91 ± 0.13	25.6	30.46 ± 0.52	1.7	167.62 ± 2.89	45.48 ± 0.81
16C	420	54	4.01 ± 0.18	43.7	10.99 ± 0.35	2.3	37.8 ± 1.84	12.1 ± 0.51
	530	54.5	4.39 ± 0.19	43.5	11.39 ± 0.37	2	38.15 ± 1.11	11.79 ± 0.36
18C	420	60.6	3.37 ± 0.15	37.7	8.66 ± 0.37	1.7	27.46 ± 1.41	8.26 ± 0.39
	530	56	3.18 ± 0.15	42.2	8.25 ± 0.31	1.8	28.45 ± 2.05	8.46 ± 0.51

The average lifetime for three exponential decay were calculated using the following relation[2]

$$\tau_{avg} = \frac{A_1\tau_1^2 + A_2\tau_2^2 + A_3\tau_3^2}{A_1\tau_1 + A_2\tau_2 + A_3\tau_3}$$

Note that the τ_{avg} calculated from the blue and broadband emission peak maxima show only slight differences, which denotes a balance between excited states.[3, 4] In both cases, there is a second recombination channel and the exciton populations for both channels are thermally coupled, which explains the similarity of the decay traces measured at different wavelengths.[4]

Table S7. Analysis of the PL time decay profiles presented in Figure S5 for all the synthesized crystals. k_r and k_{nr} are the radiative and non-radiative recombination rates, respectively.

RPLP Platelets	PL peak, nm	τ_{avg}, ns	$PLQE$, %	K_r, MHz	K_{nr}, MHz
UDA	395	3.06	2	6.53	319.91
	406	3.2		6.25	306.1
	525	3.42		5.85	286.51
1-MDA	412	16.53	3	1.82	58.7
	590	17.56		1.71	55.23
N-MDA (10C)	425	37.16	3	0.81	26.11
	525	38.70		0.78	25.06
12C	430	46.12	9	1.74	19.95
	525	46.15		1.74	19.93
14C	430	45.19	11	2.44	19.7
	525	45.48		2.42	19.57
16C	420	12.1	2.5	2.07	80.61
	530	11.79		2.13	82.69
18C	420	8.26	3.5	4.24	116.88
	530	8.46		4.14	114.11

The k_r and k_{nr} can be calculated from τ_{avg} and φ_{avg} values using the following relation[2]

$$PLQE = \frac{k_r}{k_t} = \frac{k_r}{k_r + k_{nr}}$$

$$\tau_{avg} = \frac{1}{k_t}$$

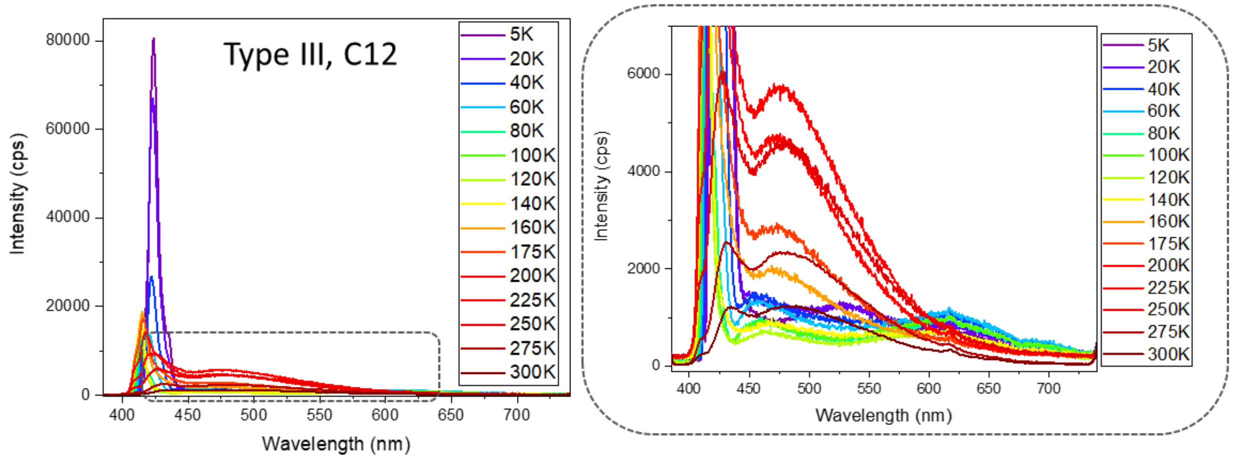

Figure S10. Temperature dependence PL spectra collected from type III, C12 from 300 K to 5 K.

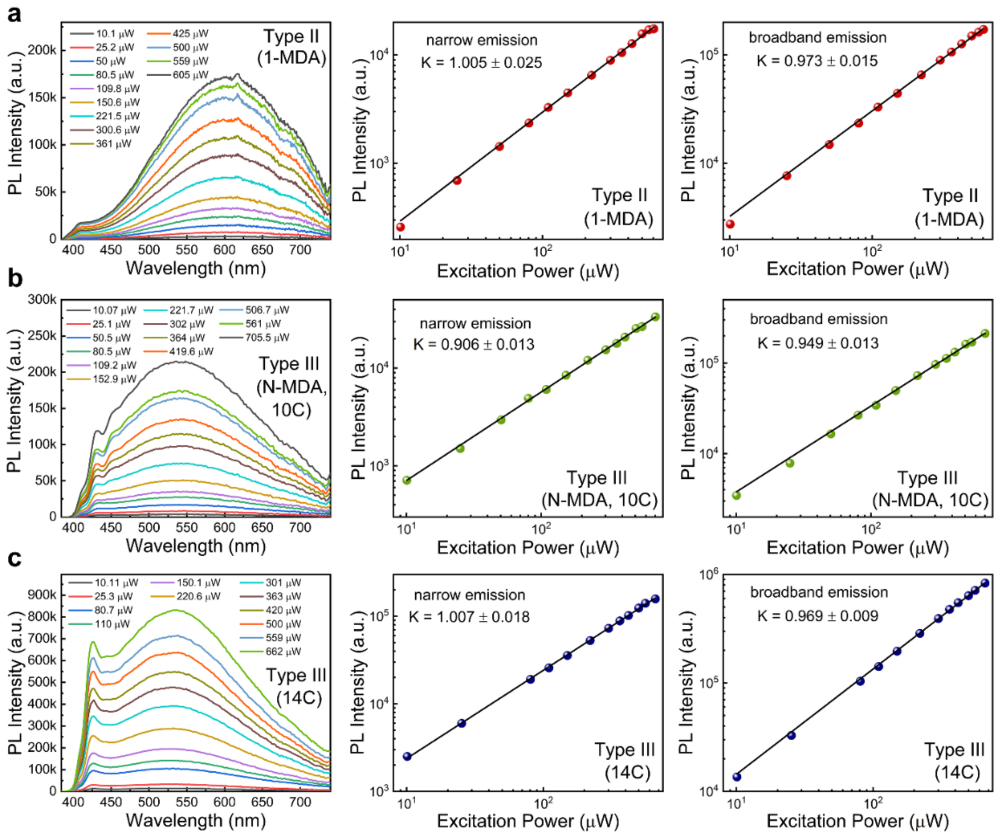

Figure S11. Excitation power-dependent PL (left) for 375 nm excitation collected from (a) type II $(1\text{-}MDA)_2PbBr_4$ (b) type III (C10) $(N\text{-}MDA)_2PbBr_4$ (c) type III (C14) $(N\text{-}MTDA)_2PbBr_4$ crystals and their corresponding PL intensity as function of excitation power in double logarithmic scale for the narrowband (middle) and broadband emission (right) fitted by using the power-law equation. The excitation spot size was approximately 1 mm diameter.

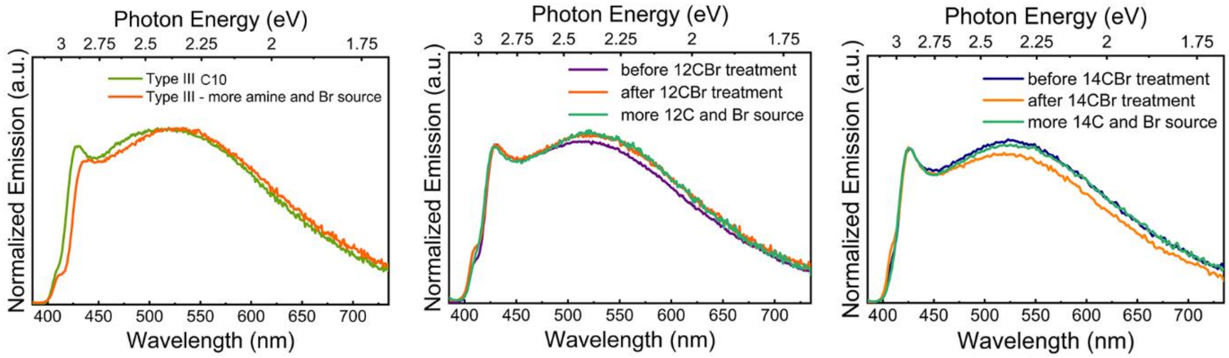

Figure S12. PL spectra of $(N\text{-}MDA)_2PbBr_4$ (left), $(12C)_2PbBr_4$ (center), and $(14C)_2PbBr_4$ (right) white-emitting platelets passivated by increasing the amine and Br content sources in the synthesis for all of them, as well as by immersing the crystals overnight in a 12CBr and 14CBr solution in methanol (50 mg/mL) in the case of the 12C and 14C type III samples, respectively. The broadband emission profile is fully preserved in all the samples, with only slight changes on its contributions with respect to the emission peak observed in the blue region.

Table S8. Octahedral mean quadratic elongation parameter, λ_{oct}, and Pb-Br-Pb angles obtained from the computed structures shown in Figure 3 of the main manuscript. We used the bulk cubic structure of $MAPbBr_3$ crystals as a reference.

	Bulk cubic $MAPbBr_3$	Computed Type I-4C	Computed Type II-4C	Computed Type III-4C	Experimental Type III-12C
λ_{oct}	1	1.016	1.025	0.996	1.009
Pb-Br-Pb angles	180°	151°	161°	176°	166°

The distortion in the Pb-Br-Pb angle appearing in the experimental structure takes place also out-of-plane, whereas the simulated structures show mainly distortions in plane.

The relaxed cells keep an almost tetragonal structure, although some tilting appears, especially in the type II one, as shown in Table S9.

Table S9. Cell parameters obtained for the relaxed computed structures based on an example of type I with 4C in their aliphatic chain. Type I parameters are reported from Ref. [5].

	a	b	c	α	β	γ
Computed Type I-4C	8.25460	8.14220	27.46310	90	90	90
Computed Type II-4C	8.91552	7.78136	26.21518	91.0271	90.1007	90.3503
Computed Type III-4C	8.27451	8.34155	23.88864	91.1398	89.8136	90.0022

The shortening of the *c* cell parameter is due to the shortening of the chain that effectively separates two neighbouring layers, while the binding site increases in size. It is interesting to notice that in the type II crystals, the *a* and *b* cell parameters are very different, as the chain tilts tend to align along the *a* axis, probably to reduce the steric hindrance.

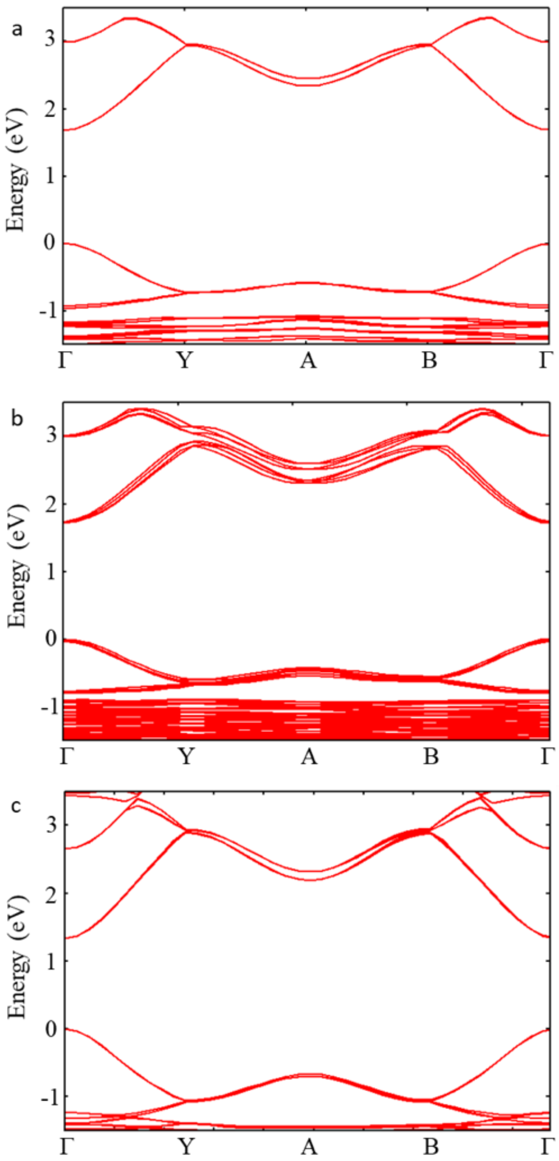

Figure S13. Band structures associated to the computed compounds using a) type-I, b) type II and c) type-III amines with band gap respectively of 1.7 eV, 1.7eV and 1.6eV. The valence and conduction bands are mainly constituted by Br-5$p_x^2p_y^2$ orbitals (together with Pb-6s^2) and by Pb-6$p_x^0p_y^0$, respectively,[6] therefore the bandgap and optical properties are largely affected by the in-plane distortions.

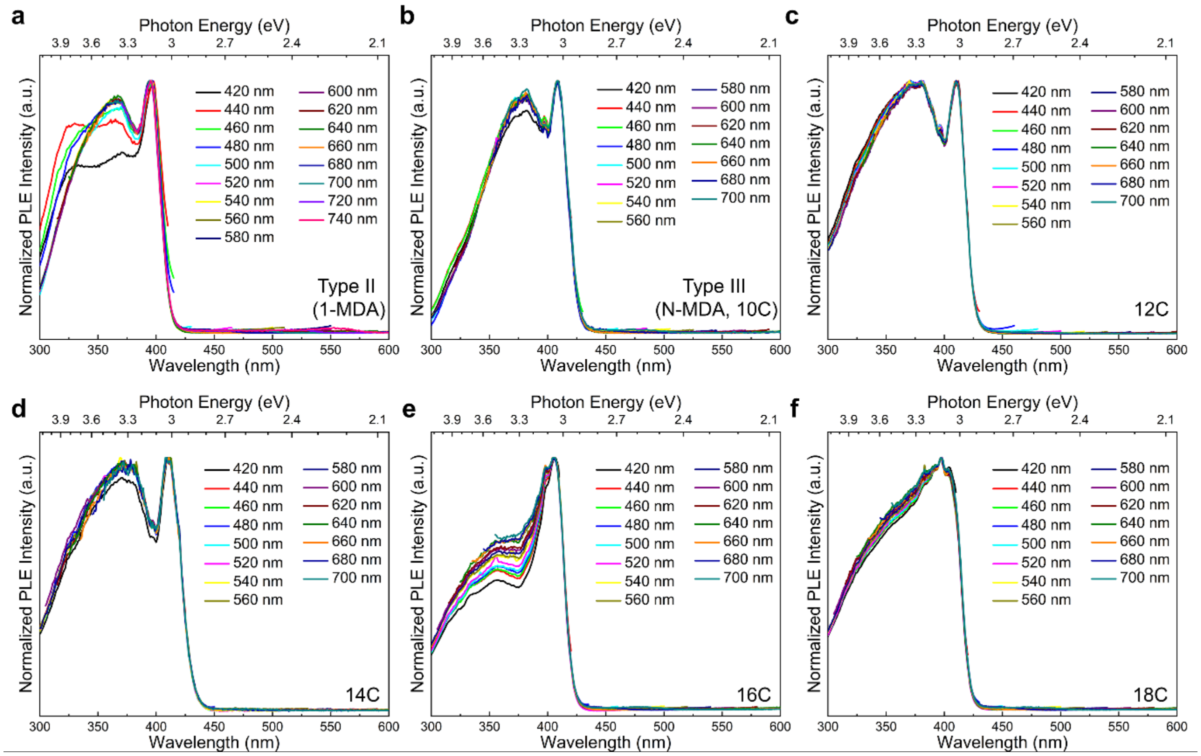

Figure S14. PLE spectra of the synthesized crystals that exhibited broadband emission acquired at different emission wavelengths. (a) $(1\text{-MDA})_2PbBr_4$, (b) $(N\text{-MDA})_2PbBr_4$, (c) $(12C)_2PbBr_4$, (d) $(14C)_2PbBr_4$, (e) $(16C)_2PbBr_4$, (e) $(18C)_2PbBr_4$. All the spectra demonstrated identical shapes and features.

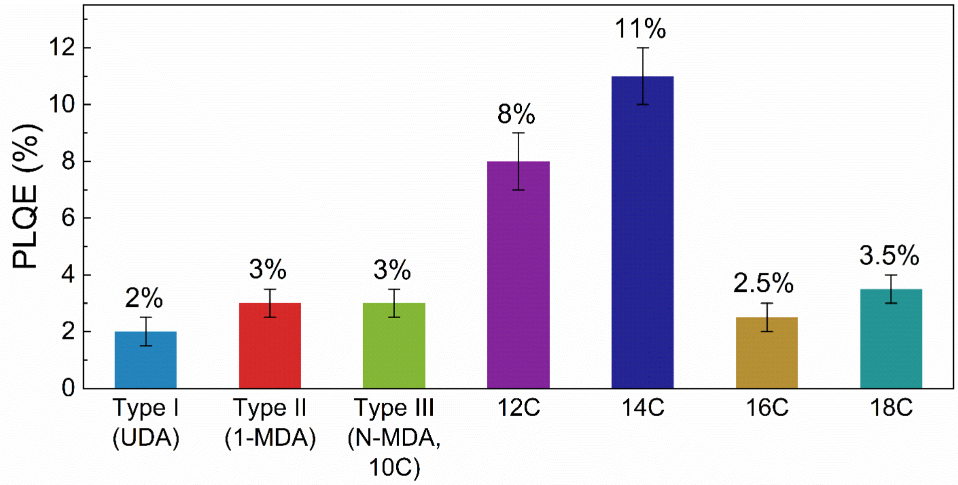

Figure S15. Average PLQE values measured from the synthesized RPLP; the calculated standard deviations are shown by the vertical bars. Crystals prepared by employing 12C and 14C organoamines show comparatively higher $PLQE$ values.

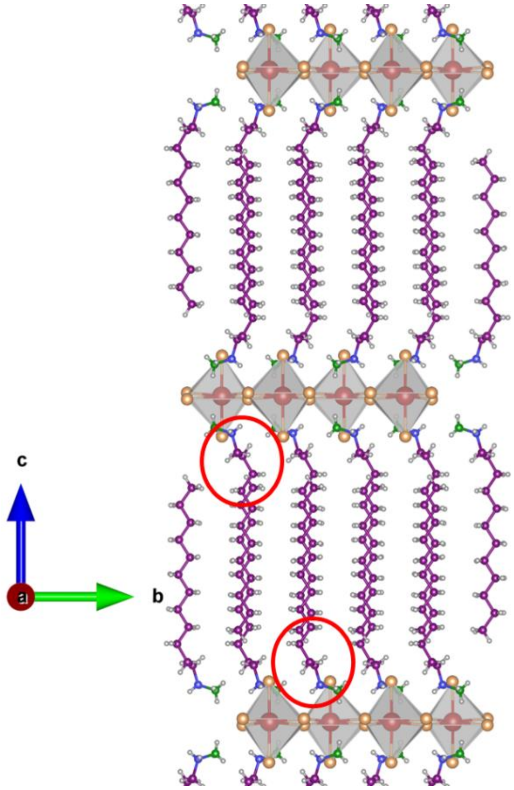

Figure S16. A view along *c* axis of the crystal packing of the (N-MDDA)$_2$PbBr$_4$ platelets showing the bending of the chains (framed in red) in the vicinity of the anchor site.

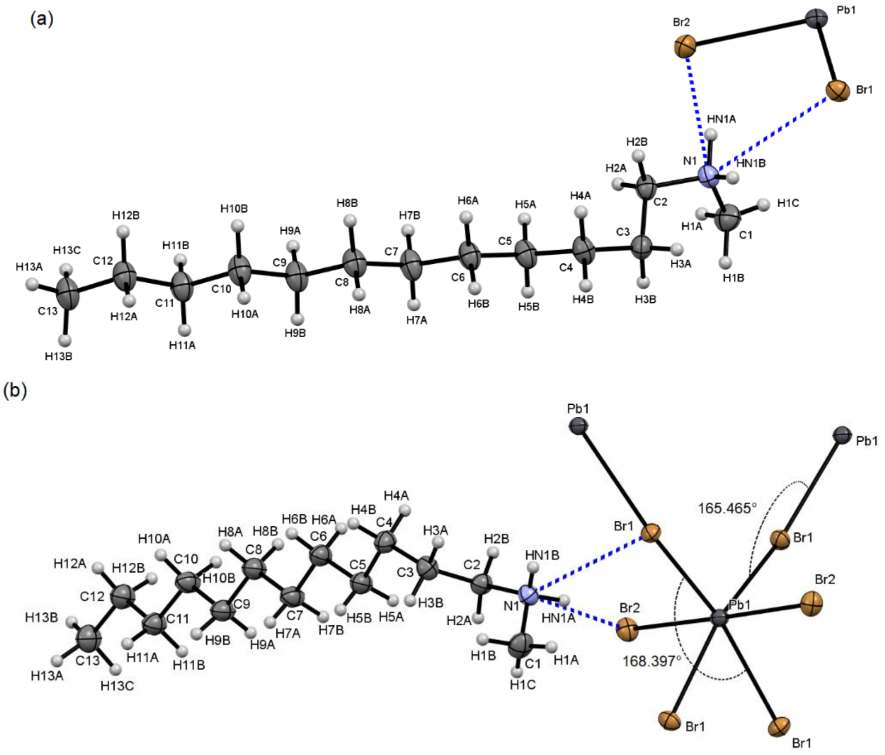

Figure S17. A view of the asymmetric unit with the atomic labelling scheme (a). A view of the asymmetric unit plus two symmetry equivalent Pb atoms and four symmetry equivalent Br atoms (b) to complete the octahedron and to show the distortion of the inorganic layer [the Pb–Br–Pb angle is 165.465 (15)° and the Br–Pb–Br angle is 168.397(16)°, see Table S3]. The broken blue lines in (a) and (b) indicate two of the N—H···Br hydrogen bonds listed in Table S4.
Ellipsoids are drawn at 50% of probability level.

Table S10. Crystal data, data collection, and structure refinement details for the $(N\text{-}MDDA)_2PbBr_4$ platelets. Data collected at 100 K.

Crystal data	
Chemical formula	$C_{26}H_{60}Br_4N_2Pb$
M_r	927.59
Crystal system, space group	Orthorhombic, *Pbcn*
Temperature (K)	100
a, *b*, *c* (Å)	8.1130 (3), 8.9440 (4), 47.6990 (6)
V (Å^3)	3461.2 (2)
Z	4
Radiation type	Synchrotron, λ = 0.72931 Å
μ (mm^{-1})	10.09
Crystal size (mm)	0.05 × 0.05 × 0.01
Data collection	
Diffractometer	Multi-axis PRIGo goniometer
Absorption correction	Empirical (using intensity measurements)
T_{min}, T_{max}	0.240, 0.746
No. of measured, independent and observed [$I > 2\sigma(I)$] reflections	55985, 4585, 4165
R_{int}	0.062
$(\sin \theta/\lambda)_{max}$ (Å^{-1})	0.719
Refinement	
$R[F^2 > 2\sigma(F^2)]$, $wR(F^2)$, S	0.029, 0.074, 1.07
No. of reflections	4585
No. of parameters	240
H-atom treatment	Only H-atom coordinates refined
	$w = 1/[\sigma^2(F_o^2) + (0.0288P)^2 + 11.8791P]$ where $P = (F_o^2 + 2F_c^2)/3$
$\Delta\rangle_{max}$, $\Delta\rangle_{min}$ (e Å^{-3})	1.01, -2.55

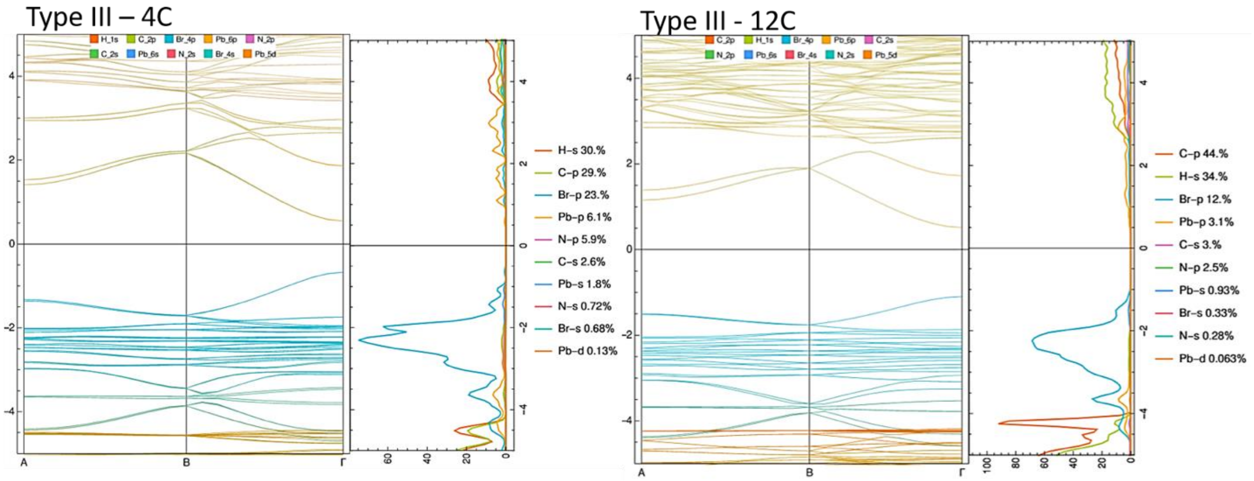

Figure S18. Band structure and DOS of the calculated type III-4C structure and the experimental (N-MDDA)$_2$PbBr$_4$ structure. While the nature and the dispersion of the bands does not change, the bandgap is reduced by 0.5 eV, thus making the use of C4 adequate exclusively for the study of the interactions between the N-H groups and the Br anions, as distortions due to the chains are not accounted for.

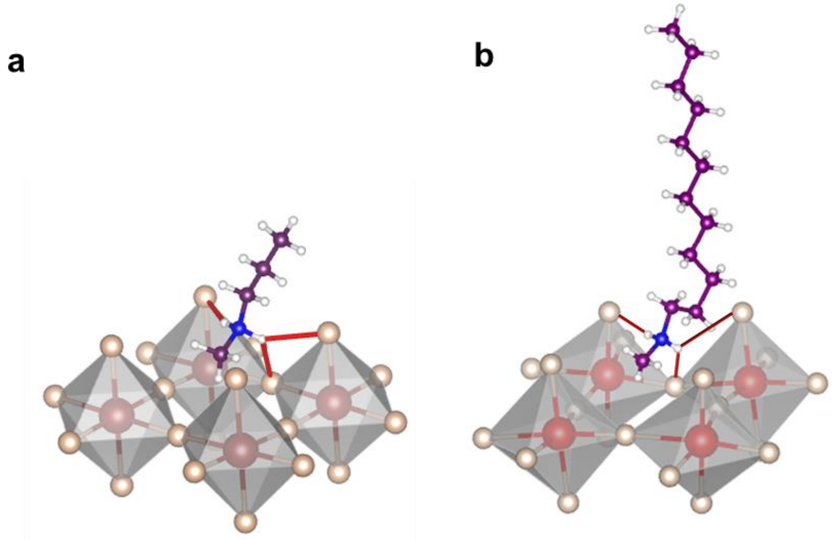

Figure S19. Close view of (a) the simulated (C4) and (b) the experimental (C12) H-binding geometry for type III structure.

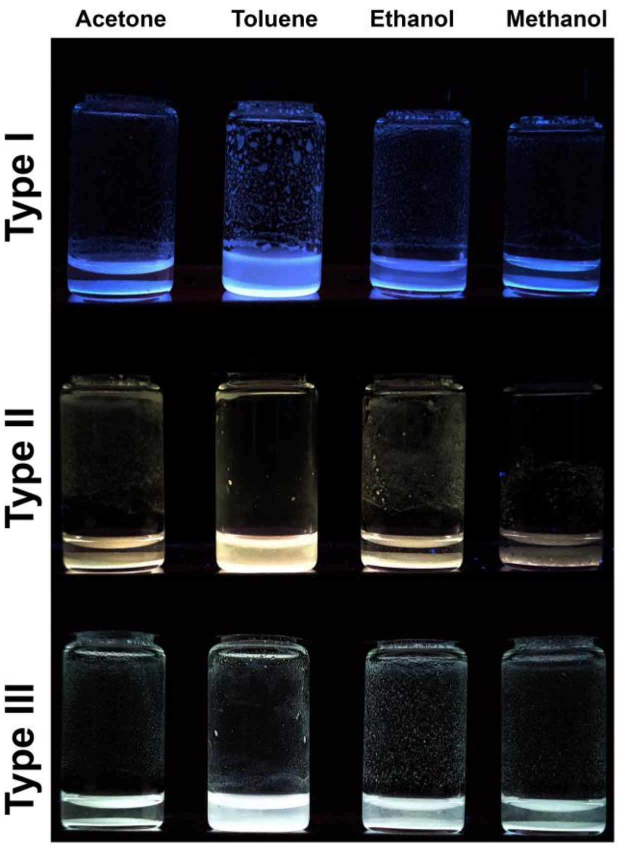

Figure S20. Picture under UV light of the vials containing the synthesized RPLP powders dispersed in different solvents.

References

[1] B. Dhanabalan, A. Castelli, M. Palei, D. Spirito, L. Manna, R. Krahne, M. Arciniegas, Nanoscale 2019, 11, 8334.

[2] J. R. Lakowicz, *Principles of fluorescence spectroscopy*, Springer science & business media, 2013.

[3] Z. Yuan, C. K. Zhou, J. Messier, Y. Tian, Y. Shu, J. M. Wang, Y. Xin, B. W. Ma, Adv. Opt. Mater. 2016, 4, 2009; H. Shao, X. Bai, H. Cui, G. Pan, P. Jing, S. Qu, J. Zhu, Y. Zhai, B. Dong, H. Song, Nanoscale 2018, 10, 1023; E. R. Dohner, A. Jaffe, L. R. Bradshaw, H. I. Karunadasa, J. Am. Chem. Soc. 2014, 136, 13154.

[4] B. Febriansyah, T. Borzda, D. Cortecchia, S. Neutzner, G. Folpini, T. M. Koh, Y. Li, N. Mathews, A. Petrozza, J. England, Angew. Chem. Int. Ed. 2020, 59, 10791.

[5] X. Gong, O. Voznyy, A. Jain, W. Liu, R. Sabatini, Z. Piontkowski, G. Walters, G. Bappi, S. Nokhrin, O. Bushuyev, M. Yuan, R. Comin, D. McCamant, S. O. Kelley, E. H. Sargent, Nat. Mater., 2018, 17, 550.

[6] Y. Cho, T. C. Berkelbach, J. Phys. Chem. Lett., 2019, 10, 6189.
